# Parameters’ Influence on Measured Behavior of Rebar Reinforced UHPC in Tension

**DOI:** 10.3390/ma19194247

**Published:** 2026-10-08

**Authors:** Yuchen Zhang, Sumei Zhang, Xianzhi Luo, Chaofan Wang, Guojun Wang

**Affiliations:** 1 School of Intelligent Civil and Ocean Engineering, Harbin Institute of Technology, Shenzhen, University Town, Shenzhen 518055, China; 2 Guangdong Provincial Key Laboratory of Intelligent and Resilient Structures for Civil Engineering, Harbin Institute of Technology, Shenzhen, Shenzhen 518055, China

**Keywords:** rebar reinforced UHPC (R-UHPC), specimen thickness, gauge length, reinforcement ratio, rebar configuration, tensile behavior

## Abstract

Direct tensile testing serves as the principal approach for assessing the mechanical performance and cracking behavior of rebar reinforced ultra-high-performance concrete (R-UHPC). However, the measured outcomes are notably sensitive to specimen dimensions and rebar configuration. This study investigates the effects of specimen thickness (50 and 100 mm), gauge length (70 and 170 mm), reinforcement ratio (1.1–6.2%), and rebar layout (a single rebar and distributed rebars) on the tensile response of R-UHPC through direct tensile tests. A total of 18 R-UHPC dog-bone specimens were tested. The UHPC had a steel fiber volume fraction of 2.0% and tensile strengths ranging from 10.9 to 13.7 MPa, while Digital Image Correlation (DIC) was used to capture the full-field crack evolution and deformation process. The test results reveal that R-UHPC exhibits an effective influence zone around rebar. In thick specimens, edge regions beyond the effective influence zone suffer from intensified strain localization, reducing effective tensile strength of the UHPC component to approximately 0.69 times that of plain UHPC. Within the effective influence zone, the reinforcement ratio influenced the UHPC component strength contribution, with the effective tensile strength decreasing from 0.99 times that of plain UHPC at a 1.1% reinforcement ratio to 0.82 times at 3.1%, and further to 0.53 times at 6.2%. Regarding failure modes, R-UHPC demonstrates a consistent transition from pre-peak multiple cracking to post-peak strain localization at the main crack. Increasing the gauge length averages this localized deformation and reduces the measured elongation at fracture. For specimens with the same gauge length, the yielded rebar length (*L*_p_), which governs the extent of localized deformation, increases with rebar diameter and can be expressed as *L*_p_ = 0.011 *f*_y_ *d*_b_. The coefficient 0.011 is approximately half the empirical value of 0.022, which is commonly adopted for strain penetration in plastic hinge regions of conventional reinforced concrete members. The distributed rebar layout in thick specimens enlarges the effective influence zone, improving cross-sectional strain homogeneity and enhancing the UHPC component effective tensile strength relative to a single rebar layout. At the same time, the associated reduction in rebar diameter intensifies strain localization and decreases elongation at fracture.

## 1. Introduction

Ultra-high-performance concrete (UHPC) exhibits ultrahigh compressive strength, tensile strain-hardening behavior, and excellent durability, making it highly attractive for civil engineering structures exposed to aggressive service environments and demanding performance requirements [1,2,3,4]. In engineering practice, UHPC is typically reinforced with steel rebars to form rebar reinforced UHPC (R-UHPC) members, allowing its material advantages to be effectively utilized while satisfying structural demands for load-bearing and deformation capacities [5,6,7]. Owing to its high material cost, UHPC is seldom used as a full independent structural member; instead, it is commonly applied as thin layers, local strengthening layers, or composite components in critical load-bearing regions or durability-vulnerable zones [8,9,10,11]. Typical applications include the negative-moment regions of bridge decks, where thin UHPC layers are commonly cast on the top surface of composite bridge decks or deck overlays [12,13,14,15]. Jiang et al. [16] investigated the flexural behavior of deck overlays incorporating a UHPC top layer and reported that the UHPC layer increased the cracking resistance of the members by 150–275%, substantially improving their crack control capacity. For the strengthening of ordinary concrete members, Jiang and Li et al. [17] studied the mechanical behavior of UHPC strengthened concrete beams under positive and negative bending moments, showing that UHPC placed on the tensile side can delay cracking and reduce the number of cracks, whereas UHPC placed on the compression side can provide greater load-bearing and deformation reserves at the ultimate stage. For local repair of existing structures, Safdar et al. [18] and Luo et al. [19] reported that R-UHPC repair layers can enhance the stiffness of concrete beams and postpone crack formation. In these applications, the ultrahigh compressive strength of UHPC generally provides a substantial safety margin, whereas the tensile performance of R-UHPC is more likely to govern the long term service behavior of the member. This tensile performance is directly associated with crack initiation, crack propagation, and crack-width evolution, and may further affect the durability and load-bearing performance of the member. Therefore, reliable characterization of the tensile performance of R-UHPC is essential for its safe and rational application in critical structural regions.

R-UHPC tensile behavior is commonly characterized by direct tension tests, which enable the load–deformation response, crack evolution, and failure mode of specimens to be directly assessed under tensile loading [20,21,22,23]. Qiu et al. [24] showed that increasing the reinforcement ratio can restrain maximum crack width development and increase both yield and ultimate loads. Luo et al. [9] employed digital image correlation (DIC) to monitor crack development in R-UHPC, clarifying the effects of fiber content and reinforcement ratio on crack number, crack spacing, and maximum crack width. Guo et al. [25] combined direct tension tests with acoustic emission monitoring to investigate R-UHPC with low fiber contents and found that high-strength rebars could induce longitudinal cracking at the failure stage, thereby improving the overall tensile ductility of the specimens. Huang et al. [26] further reported that steel fibers enhanced the UHPC-rebar bond strength and intensified strain concentration in the embedded rebars, thereby reducing the overall deformation capacity of the specimens; nevertheless, increasing the reinforcement ratio effectively improved their tensile ductility. These studies indicate that existing direct tension investigations on R-UHPC have mainly focused on load-bearing capacity, deformation capacity, and crack control performance, with key evaluation indices including peak load, peak strain, crack number, crack spacing, and crack width.

Despite the use of common evaluation indices, considerable discrepancies remain among the direct tension test results reported for R-UHPC. Aghdasi et al. [27] tested R-UHPC specimens with a 101.6 mm × 101.6 mm cross-section, a 355.6 mm gauge length, and a single centrally placed rebar, and reported an elongation at fracture of only 6.0% at a fiber volume fraction of 2.5% and a reinforcement ratio of 2.8%, accompanied by pronounced strain localization. In contrast, Zhang et al. [28] obtained an elongation at fracture of up to 25.4% using specimens with a smaller cross-section of 50 mm × 50 mm and a shorter gauge length of 70 mm under comparable fiber content, reinforcement ratio, and single rebar layout, indicating elongation close to that of bare steel rebars. The substantially different elongation responses reported in these two studies may be partly attributed to the geometric averaging effect over different gauge lengths. Differences are also evident in the effective utilization of the tensile strength of UHPC when assessing the load-bearing capacity of R-UHPC, as reflected by the UHPC strength reduction factor. Luo et al. [29] obtained a UHPC strength reduction factor of 0.80 using specimens with a 100 mm × 50 mm cross-section, a 150 mm gauge length, and three uniformly distributed rebars, whereas Bian [30] reported a value of approximately 0.54 for specimens with the same cross-sectional size and reinforcement ratio but only a single centrally placed rebar. The comparison between these studies demonstrated that the effective tensile contribution of the UHPC component may vary with rebar configuration. Shi et al. [31] further investigated the effects of rebar number and diameter on dog-bone-shaped R-UHPC specimens, with emphasis on peak tensile load-bearing capacity. Building on these studies, the present work investigates the effects of gauge length, specimen thickness, reinforcement ratio, and rebar layout on the tensile response of R-UHPC specimens throughout the loading process from initial loading to fracture, with particular emphasis on crack evolution, deformation process, and the variation pattern of the UHPC component strength reduction factor with specimen parameters.

To investigate the effects of specimen parameters on the measured tensile behavior of R-UHPC, a series of tensile specimens varying in gauge length, cross-sectional thickness, reinforcement ratio, and rebar layout were designed and subjected to direct tension tests. The digital image correlation (DIC) technique was employed to monitor the full-field cracking and deformation evolution processes, while the load-strain response was simultaneously recorded. Based on the experimental data, the cracking characteristics, load-bearing capacity, and deformation capacity were quantitatively analyzed. By comparing the tensile response of R-UHPC specimens with different parameters, the effects of gauge length and cross-sectional thickness on the mechanical performance of specimens are evaluated. Furthermore, the roles of reinforcement ratio and layout in mitigating strain localization and controlling crack widths are clarified. Ultimately, this study provides an experimental basis for understanding the effects of specimen parameters on the measured tensile behavior of R-UHPC and interpreting direct tension test results.

## 2. Experimental Program

### 2.1. Specimen Design

**Figure 1:**
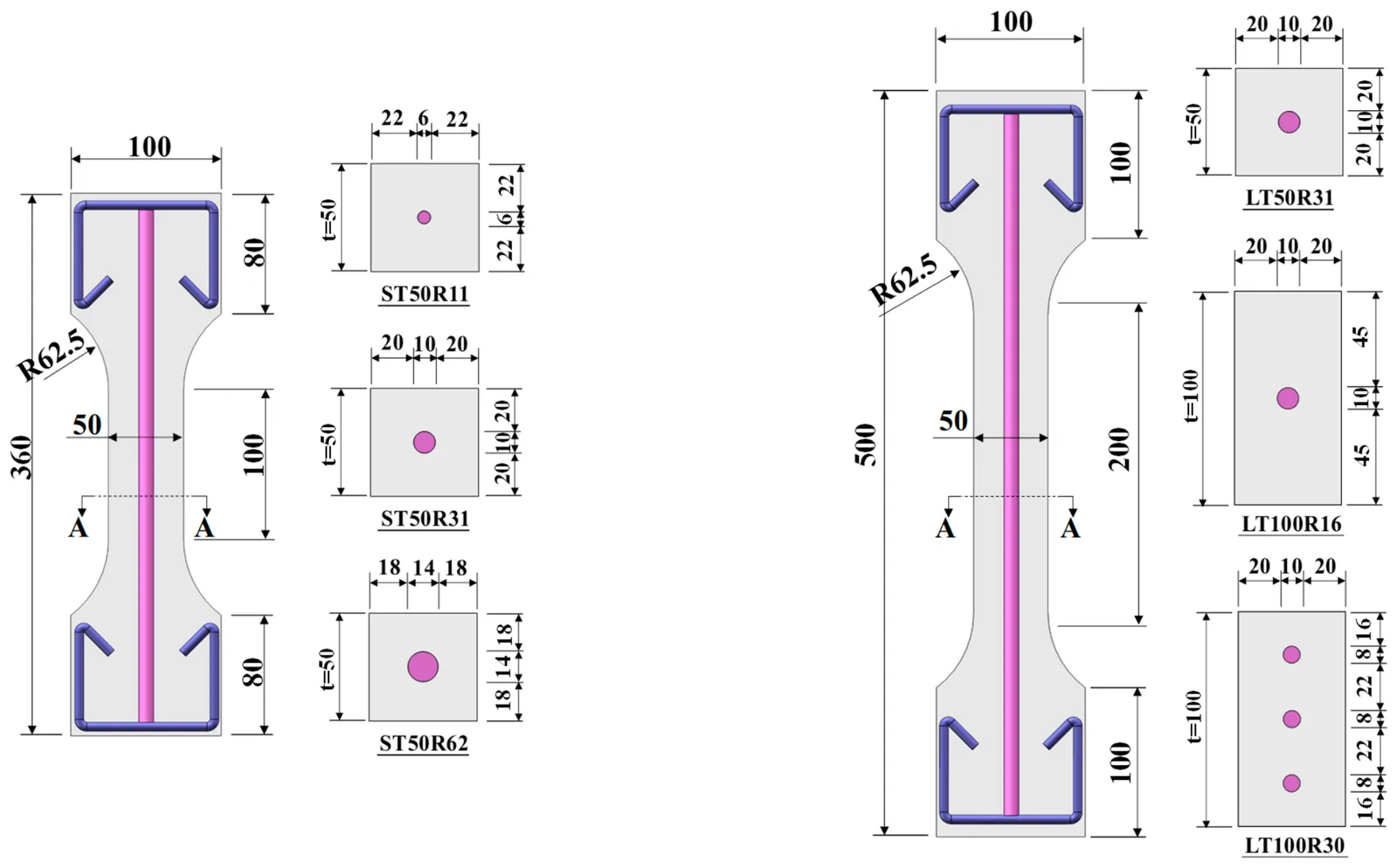
Diagram of R-UHPC specimens [28].

Dog-bone shaped specimens have been widely adopted for determining the tensile properties of UHPC [32,33,34,35,36] and have been incorporated into recognized standards, including the Swiss standard SIA 2052 [37] and Chinese standard T/CBMF 37-2018 [33]. Featuring enlarged gripping ends, this geometry helps reduce stress concentrations at the grips and promotes failure within the gauge length, thereby enabling the reliable assessment of tensile stress–strain responses [38,39,40]. Accordingly, dog-bone shaped tension specimens (Figure 1) were employed in this study to evaluate the tensile behavior of R-UHPC. A total of 18 R-UHPC specimens from 6 groups were analyzed, including 15 new specimens and 3 previously reported ST50R31 specimens together with their corresponding load–strain curves from Ref. [28]. The specimen preparation and loading procedures were consistent with those adopted for the new specimens. The detailed specimen parameters are summarized in Table 1, with each group comprising 3 replicate specimens. ST50R11, ST50R31, and ST50R62 differ in reinforcement ratio, accompanied by changes in rebar diameter. ST50R31 and LT50R31 differ in gauge length. For LT50R31 and LT100R16, the rebar diameter remains unchanged, while specimen thickness and reinforcement ratio vary. LT100R16 and LT100R30 differ in rebar layout and rebar diameter.

Following the dimensional recommendations for UHPC tension specimen outlined in these standards, three types of R-UHPC specimens were designed with a constant cross-sectional width of 50 mm, while varying in gauge length and thickness. The overall dimensions of the specimens were 100 mm × 50 mm × 360 mm, 100 mm × 50 mm × 500 mm, and 100 mm × 100 mm × 500 mm, respectively. The specimens with a 50 mm thickness were each reinforced with a single steel rebar, corresponding to reinforcement ratios of 1.1%, 3.1%, and 6.2%, achieved using rebar diameters (*d*_b_) of 6 mm, 10 mm, and 14 mm, respectively. To ensure sufficient anchorage between the steel rebar and UHPC, short anchorage rebars of the same diameter with a length of 3 db were welded symmetrically to both ends of the longitudinal rebar in accordance with the Chinese standard GB 50010-2010 [41]. For the 100 mm thick specimens, two rebar layouts were considered: one with a single centrally placed rebar and the other with three distributed rebars with a clear spacing of 22 mm, corresponding to a center-to-center spacing of 30 mm.

### 2.2. Materials

The UHPC material was supplied as a commercially available proprietary product comprising premixed dry powder, steel fibers, superplasticizer, and water. The mass ratio of commercial premixed dry powder, straight steel fibers, hooked-end steel fibers, and water was 12.08:0.45:0.45:1.00, with an effective water-to-binder ratio of 8.28%. The total steel fiber content was 2.0% by volume, composed of a hybrid combination of straight and hooked-end fibers in equal proportions (1.0% each). Both fiber types had a nominal length of 16 mm and a specified minimum tensile strength of 2800 MPa; the straight fibers featured a diameter of 0.20 mm (aspect ratio of 80), and the hooked-end fibers had a diameter of 0.22 mm (aspect ratio of 72). Basic material property tests of the UHPC were conducted in accordance with the Chinese standards GB/T 31387-2015 [32] and T/CBMF 37-2018 [33]. Specifically, three 100-mm cubes were cast for compressive strength testing, three 100 × 100 × 300 mm prisms for elastic modulus determination, and three dog-bone tensile specimens with a 50 × 50 mm cross-section in the gauge region for tensile strength characterization.

The steel rebars used in the tests were commercial HRB400 bars with a nominal yield strength (*f*_y_) of 400 MPa, the tensile properties of which were tested in accordance with the Chinese standard GB/T 228.1-2021 [42]. Furthermore, to facilitate the comparison of ductility under different gauge lengths, the measured elongation at fracture was converted into equivalent values corresponding to gauge lengths of 70 mm and 170 mm by using the conversion method (Equation (1)) specified in the Chinese standard GB/T 17600.1-1998 [43]. When the rebar cross-sectional area remains constant, this conversion is expressed as:

(1) $$\delta_{r}={\left(L_{0}/L_{0r}\right)}^{0.4}\cdot \delta$$

where *L*_0_ and *L*_0r_ represent the original and reference gauge lengths, respectively; *δ* and *δ*_r_ are the elongations after fracture corresponding to *L*_0_ and *L*_0r_.

The 360 mm-long and 500 mm-long specimens were fabricated using UHPC with identical mixture proportions and rebars of the same grade; however, the materials used for the two specimen series came from different batches and exhibited differences in their measured mechanical properties. The two UHPC batches were cast in March and July, respectively. Since the specimens were cast under site conditions and transferred to the standard curing room after 2 days of curing, the differences in environmental temperatures between the casting periods may have contributed to the observed variation in tensile strength. Therefore, Table 2 and Table 3 report the material properties of the two batches separately.

### 2.3. Specimen Preparation

UHPC mixtures were prepared using a 100 L planetary mixer (Wenzhou Wengong Engineering Machinery Co., Ltd., Wenzhou, China). Prior to mixing, the inner surfaces of the mixer were dampened to prevent moisture absorption by the dry inner surfaces of the pan and ensure the effective water-binder ratio remained consistent with the design value. The mixing procedure was conducted as follows: the commercial premixed dry powder was first charged into the mixer, followed by the addition of the total quantity of mixing water and superplasticizer. After mixing for approximately 10 min, once the mixture exhibited adequate flowability, steel fibers were gradually introduced through a sieve mesh to prevent agglomeration. Mixing continued for an additional 3 min to ensure homogeneous fiber dispersion within the matrix.

**Figure 2:**
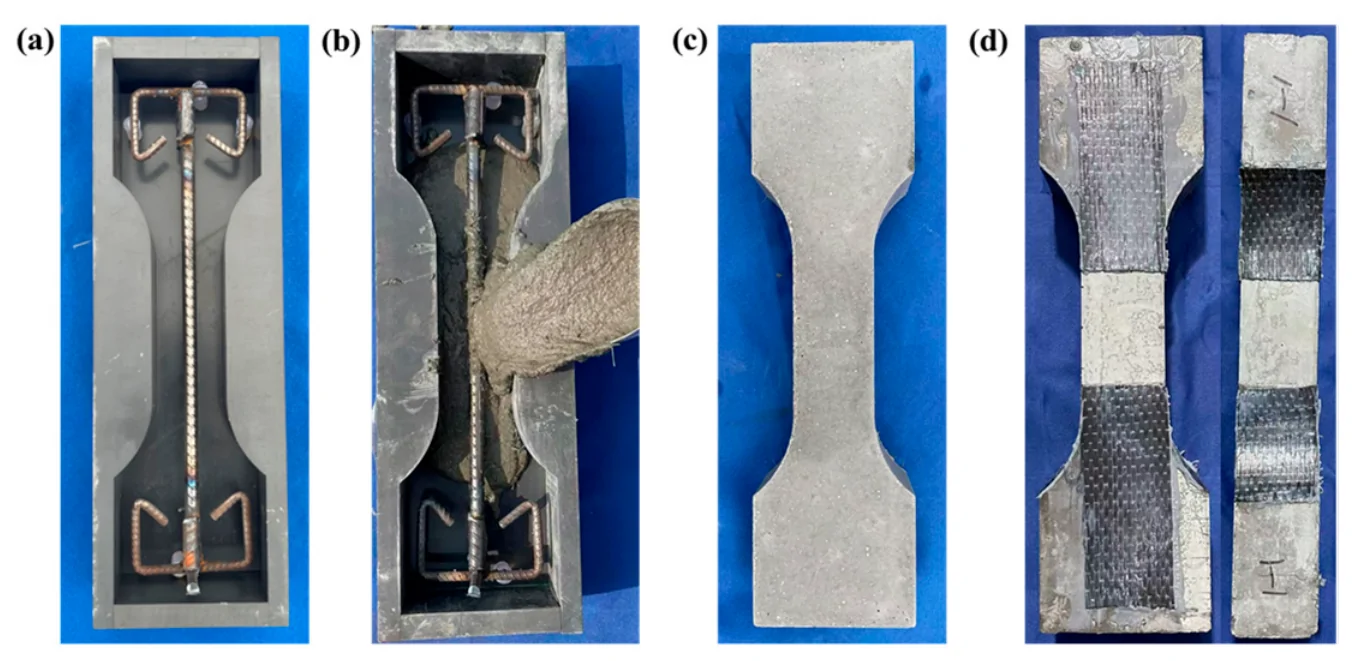
R-UHPC specimen preparation: (**a**) rebar cage positioning; (**b**) mixture casting; (**c**) cured specimen; (**d**) specimen strengthening [28].

To mitigate potential flow obstruction and non-uniform fiber distribution caused by the pre-installed rebar cage, the fresh mixture was poured to flow through the gaps between the rebar, and the mold side walls during casting. After casting, the specimens remained undisturbed for 48 h before demolding. After demolding, the specimens were transferred to a standard curing room (20 ± 2 °C; relative humidity ≥95%) and cured until the age of 28 d. Before tensile testing, the transition arcs of each dog-bone specimen were strengthened by wrapping carbon-fiber-reinforced polymer (CFRP) sheets to prevent premature failure outside the gauge region. After CFRP strengthening, the effective unstrengthened lengths of the original 100 mm and 200 mm prismatic regions shown in Figure 1 were 70 mm and 170 mm (Figure 2), respectively (Table 1). Tensile tests were conducted approximately 35 days after casting, and all R-UHPC specimens failed within the unstrengthened gauge regions.

### 2.4. Tensile Test

**Figure 3:**
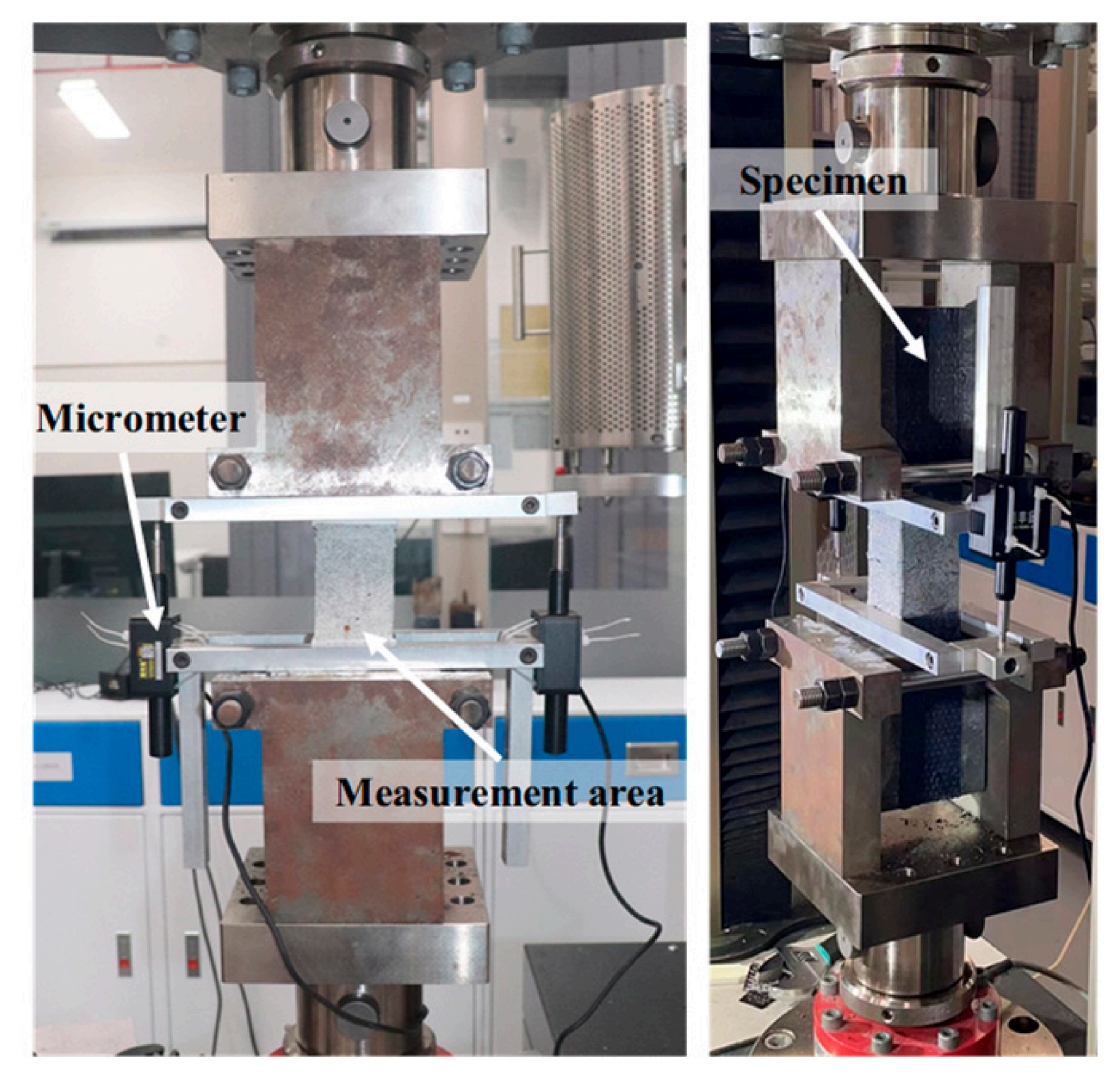
Direct tensile test setup.

Direct tensile tests (Figure 3) were performed with a digital image correlation (DIC) system to monitor the crack initiation and propagation process of the specimens throughout loading. Images with a resolution of 4240 × 2824 pixels were acquired using a 35 mm lens at a working distance of approximately 550 mm and an acquisition frequency of 0.5 fps. The specimen surface was prepared with a white background and randomly distributed black speckles with a characteristic size of approximately 0.2 mm.

Image correlation was performed using a subset size of 38 pixels and a step size of 2 pixels. The strain field was calculated using the Engineering strain tensor with a strain filter size of 5. Crack widths were extracted using virtual extensometers placed across individual cracks, with crack opening determined from the relative displacement between the two endpoints of each extensometer. The DIC measurement window was approximately 210 mm × 140 mm, corresponding to a spatial scale of approximately 0.050 mm/pixel based on the image resolution. The nominal subpixel displacement resolution of 0.01 pixel therefore corresponds to 0.00050 mm.

A rotationally fixed boundary condition was achieved at the loading end by coupling the tensile grips to the machine crosshead via end sleeves. This configuration effectively suppressed end rotation, ensuring that the observed cracking behavior reflected the realistic tensile response of the R-UHPC. Prior to tensile loading, each specimen was preloaded to 5 kN to eliminate mechanical slack, facilitate fine alignment adjustments, and minimize eccentricity. Tensile tests were then conducted under displacement control. While existing studies suggest that tensile properties of UHPC and R-UHPC can be reliably captured at rates of 0.1 mm/min to 0.5 mm/min [39,40,44], a conservative rate of 0.1 mm/min was adopted in this study to precisely capture the crack evolution process under quasi-static loading. Displacements within the gauge section were recorded simultaneously using micrometers and the DIC system, enabling cross-verification of the acquired data and ensuring the reliability of the measurements.

## 3. Results and Discussion

### 3.1. Cracking Behavior

#### 3.1.1. Cracking Process and Distribution

**Figure 4:**
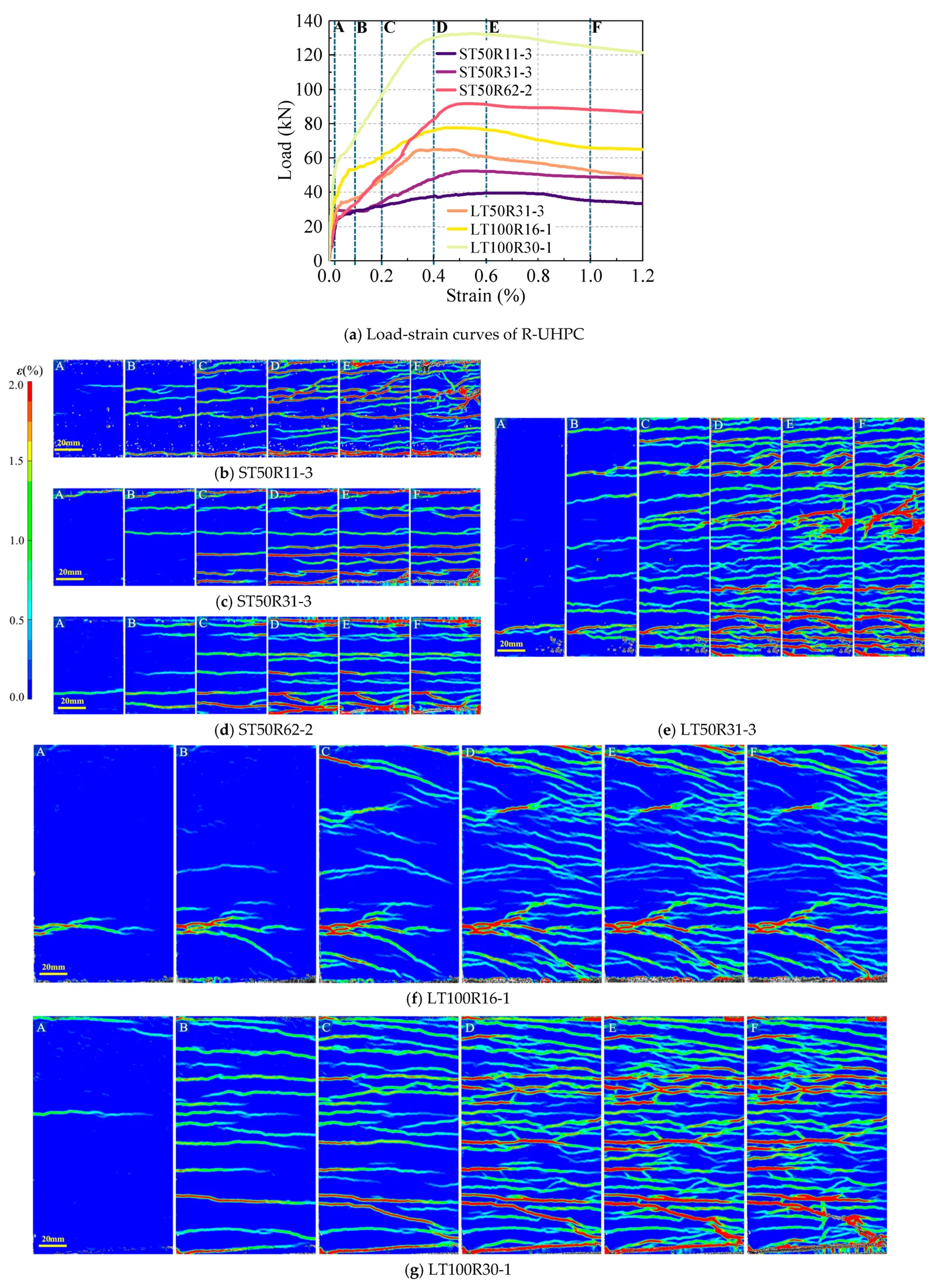
Cracking process of R-UHPC specimens.

Figure 4 presents the crack evolution of the R-UHPC specimens obtained from the maximum principal strain fields measured by DIC, in which localized regions of high principal strain correspond to crack locations. The load–displacement curves are presented together with the corresponding crack distributions at characteristic points A–F to relate the evolution of cracking to the tensile response. Across all specimens, cracking followed the same fundamental sequence. Between points A and D, cracks progressively initiated and the number of cracks increased continuously. From point D to point F, the crack number no longer increased appreciably; instead, the main crack widened rapidly, accompanied by extensive fiber pull-out, which marked the onset of degradation in load-bearing capacity. Therefore, the fundamental cracking sequence remained essentially unchanged across the investigated parameter ranges.

The combined comparison nevertheless shows that the spatial distribution of cracks was more variable in the 100 mm-thick single-rebar specimen LT100R16 (Figure 4f). Between points A and B, new cracks in the 50 mm-thick specimen LT50R31 primarily initiated independently in the regions between existing cracks, evolving into cracks extending across the visible width of the DIC measurement area with nearly uniform spacing by point B (Figure 4e). In contrast, during the same stage in LT100R16, new cracks frequently initiated in close proximity to pre-existing ones, exhibiting a crack initiation pattern similar to that reported for plain UHPC [45]. By the time the strain reached 1.0%, cracks in LT50R31 extended throughout the gauge region and exhibited an approximately symmetric distribution on the left and right sides of the measurement area. Conversely, LT100R16 contained numerous partial-width surface cracks with a pronounced asymmetric distribution, as cracks on the left side tended to be locally clustered.

The 100 mm-thick specimen LT100R30 (Figure 4g), with multiple rebars uniformly distributed across the section, still exhibited the same progressive multiple-cracking sequence as the thinner specimens and maintained a relatively uniform crack distribution. This indicates that increasing specimen thickness alone does not fundamentally alter the cracking mechanism of R-UHPC when the rebar is more uniformly distributed across the section.

In comparison, the pronounced asymmetric cracking observed in the 100 mm-thick single-rebar specimen LT100R16 suggests that the spatial distribution of rebar may play an important role in maintaining a more uniform tensile field and stabilizing crack development in thicker specimens. In the single-rebar configuration, regions farther from the rebar are less influenced by the rebar and may therefore be more susceptible to local material heterogeneity.

#### 3.1.2. Crack Density and Spacing

**Figure 5:**
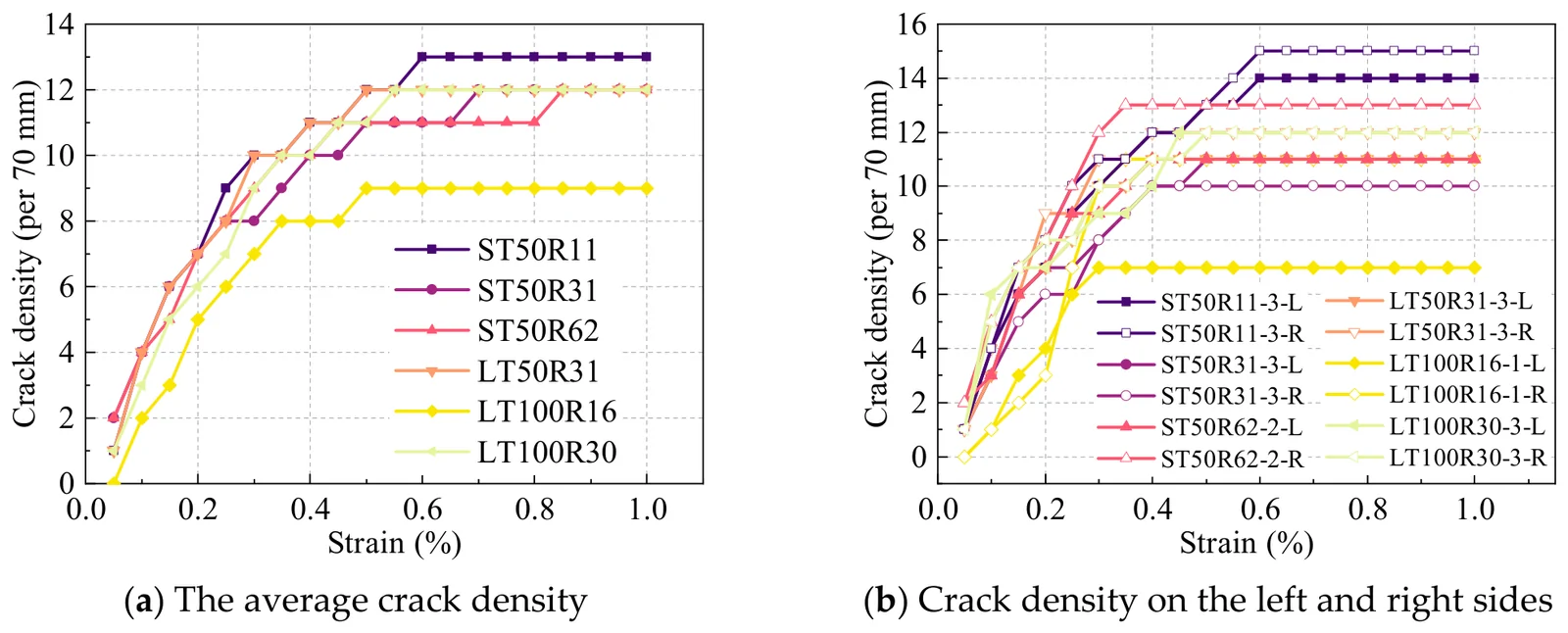
Crack density-strain curves for R-UHPC specimens.

The evolution of crack density for R-UHPC specimens with different parameters is illustrated in Figure 5. Crack counts were determined along two quarter-length lines on each side of the specimen gauge region, and their average was used as the representative crack count for each specimen. To compare the overall crack density among specimens with different gauge lengths, the crack counts of the 170 mm-gauge specimens over the entire length were converted to equivalent counts per 70 mm based on the overall average crack density. This conversion was used only to provide a common length basis for comparison and does not assume a uniform crack distribution along the gauge region. Figure 5a presents the average crack density of each specimen group, whereas Figure 5b compares the crack development on the left and right sides of representative specimens.

Despite differences in gauge length, thickness, reinforcement ratio, and rebar layout, the overall trend of crack-count development remained consistent across all R-UHPC specimens (Figure 5a). The crack count increased rapidly within the strain range of 0.0% to 0.4%; Between 0.4% and 0.7% strain, the rate of new crack formation slowed; Beyond 0.7% strain, the crack count stabilized and no further cracks formed. For example, ST50R31 developed 10 cracks by 0.4% strain and only 2 additional cracks before stabilization. The progressive reduction in the formation rate reflects the subdivision of the wider intervals between existing cracks until the multiple-cracking pattern approaches saturation.

For the 50 mm thick specimens, this stabilized crack density was essentially unchanged by gauge length or reinforcement ratio. ST50R31 and LT50R31 exhibited comparable crack density, corresponding to approximately 12 cracks per 70 mm, and ST50R11, ST50R31, and ST50R62 stabilized within a narrow range of 12–13 cracks per 70 mm despite the reinforcement ratio increasing from 1.1% to 6.2%. This saturation in crack density indicates that even 1.1% reinforcement ratio was already sufficient to trigger the characteristic multiple cracking mechanism in R-UHPC. Hence, within the investigated range, gauge length and reinforcement ratio had limited influence on the saturated crack density and did not alter the basic crack-count evolution.

The principal deviation was observed for LT100R16, whose crack density corresponded to approximately 9 cracks per 70 mm. At 1.0% strain, the crack density on the right side was about 70% higher than that on the left (Figure 5b). In contrast, LT100R30, which has the same 100 mm thickness but a distributed rebar layout, developed a stabilized average of about 12 cracks together with a much more balanced left-right evolution (Figure 5b). This contrast can be interpreted in terms of an effective influence zone around the steel rebar. Within the zone, the rebar modifies crack behavior in surrounding UHPC, promotes multiple cracking and helps control crack widths, with its influence diminishing with distance from the rebar surface. In LT100R16, the effective influence zone does not extend over the entire cross-section. Consequently, the edge regions farther from the rebar are less effectively restrained, and the development of multiple cracking becomes less uniform, resulting in a lower overall crack density. Furthermore, UHPC located outside the effective influence zone tends to behave more like plain UHPC, where crack initiation and propagation are more strongly governed by the local heterogeneity of the matrix and the spatial distribution of steel fibers. By contrast, variations in gauge length had only a limited influence on the crack density in the present tests. This indicates that the evolution and stabilized crack density in R-UHPC are governed primarily by cross-sectional characteristics, whereas gauge length mainly defines the spatial range over which cracks are observed.

**Figure 6:**
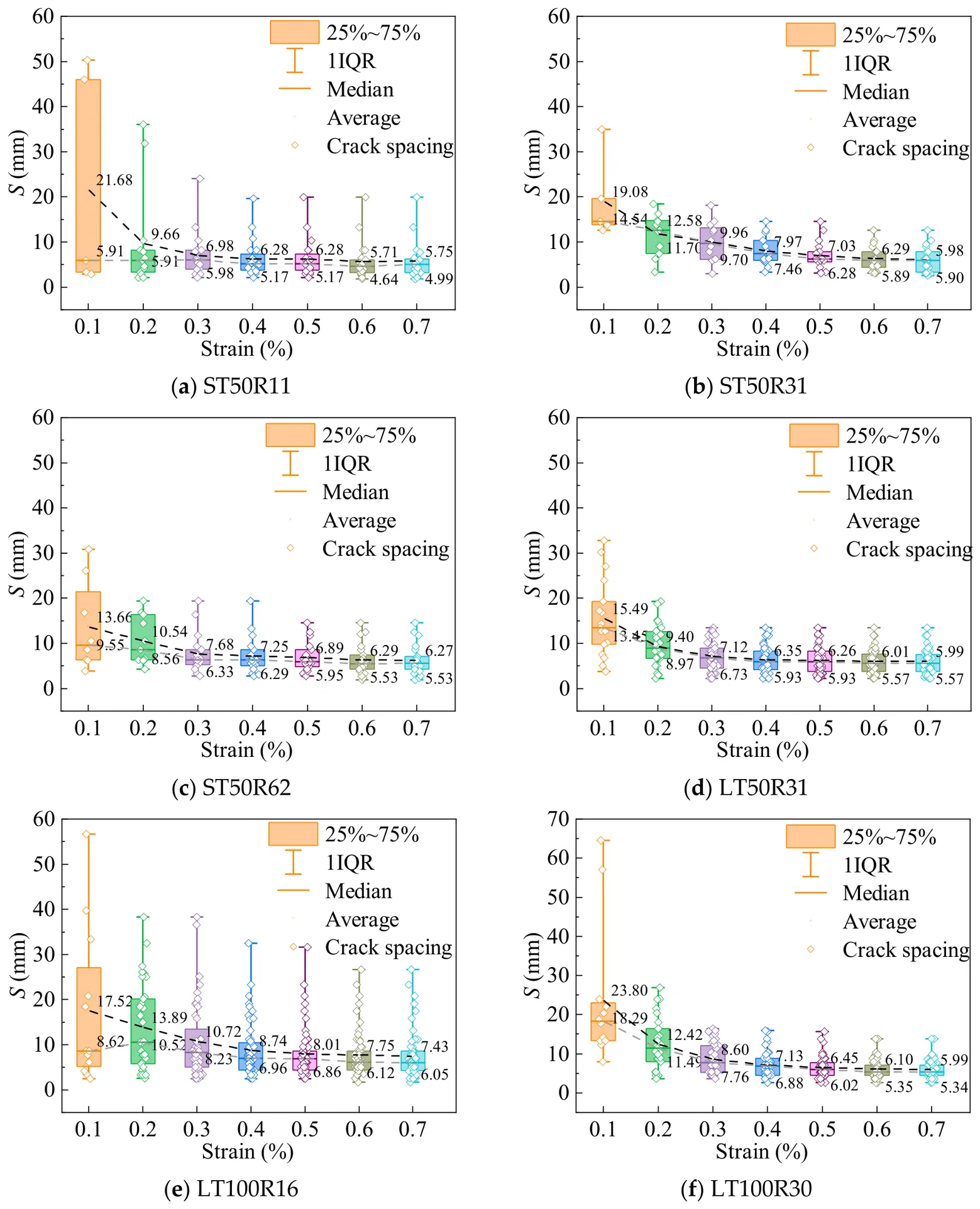
Evolution of crack spacing with strain.

Both the average and median crack spacing (*S*) of the R-UHPC specimens followed highly consistent evolutionary trends with increasing strain (Figure 6): both the mean and median crack spacing decreased rapidly during the early multiple-cracking stage and approached a stable level after approximately 0.4% strain. This evolution is the inverse counterpart of the crack-count response in Figure 5: as new cracks progressively subdivide the uncracked intervals, spacing decreases; once crack formation approaches saturation, the spacing distribution also stabilizes. The transient increase in the median spacing of LT100R16 from 0.1% to 0.2% strain is consistent with the clustered early crack initiation described in Section 3.1.1. As cracking subsequently extended into previously uncracked regions, larger intervals entered the measured spacing distribution, temporarily increasing its median.

The stabilized spacing data further clarify the effects of the investigated parameters on crack spacing. At 0.7% strain, the stabilized crack spacing values of ST50R31 and LT50R31 were comparable, suggesting that gauge length had limited influence on crack spacing within the tested specimens. Likewise, the 50 mm-thick single-rebar specimens ST50R11, ST50R31, and ST50R62 had average spacings of 5.75, 5.98, and 6.27 mm, respectively. The narrow variation in spacing suggests that, within the investigated range and at a constant steel fiber volume fraction of 2.0%, the effect of reinforcement ratio on stabilized crack spacing was limited. This finding aligns with a previous study [29], which reported that crack spacing in R-UHPC is predominantly governed by fiber-bridging action rather than reinforcement ratio.

LT100R16 retained a larger average spacing of 7.43 mm, 24.0% above that of LT50R31. The distributed-rebar specimen LT100R30 reduced the spacing to 5.99 mm, essentially restoring it to the level of the 50 mm-thick specimens. This result is consistent with the crack-count comparison and again indicates that the distinctive response of LT100R16 arises from the combination of a thick section and the spatially localized restraint associated with the single-rebar configuration.

#### 3.1.3. Crack Width Evolution

**Figure 7:**
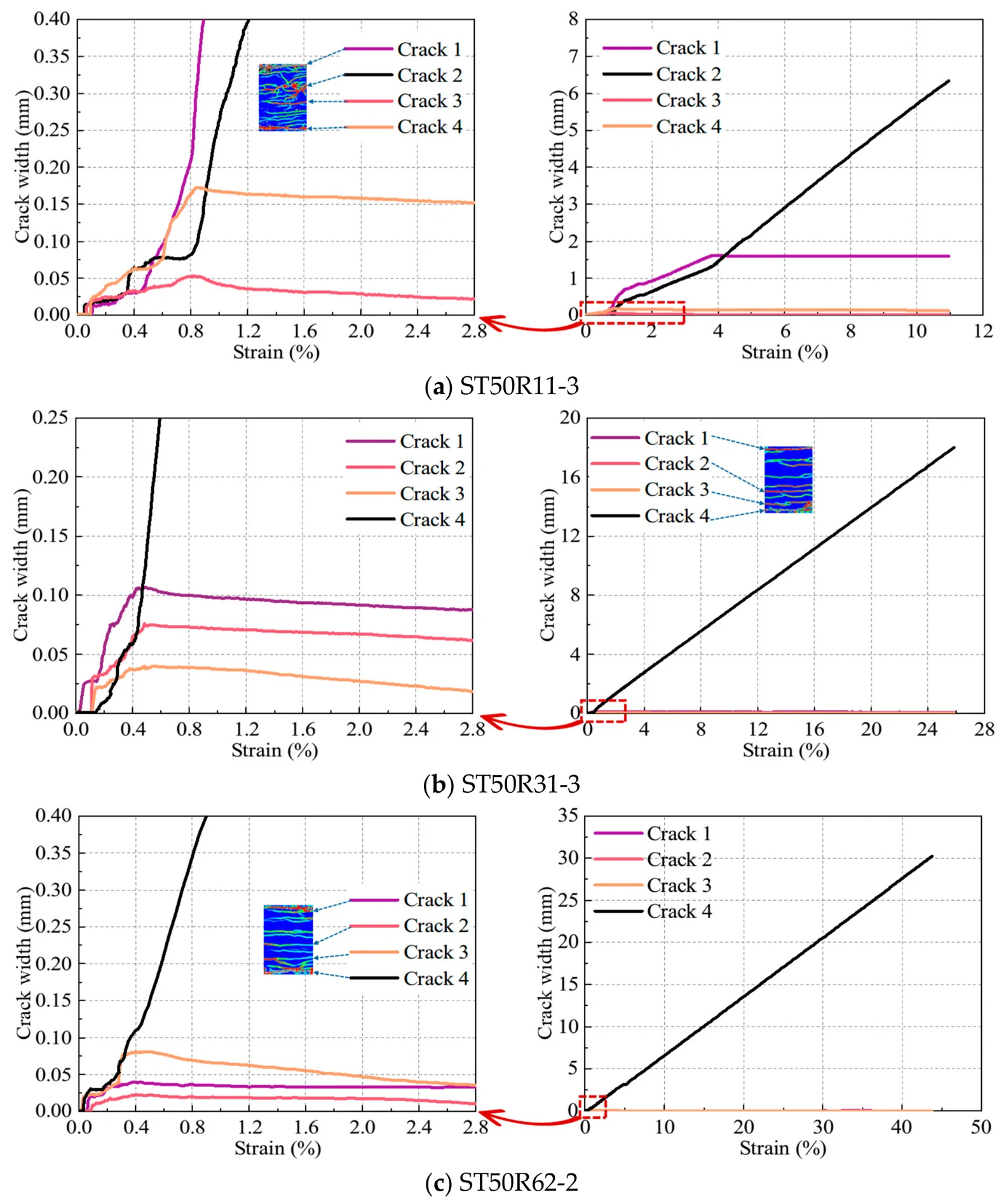

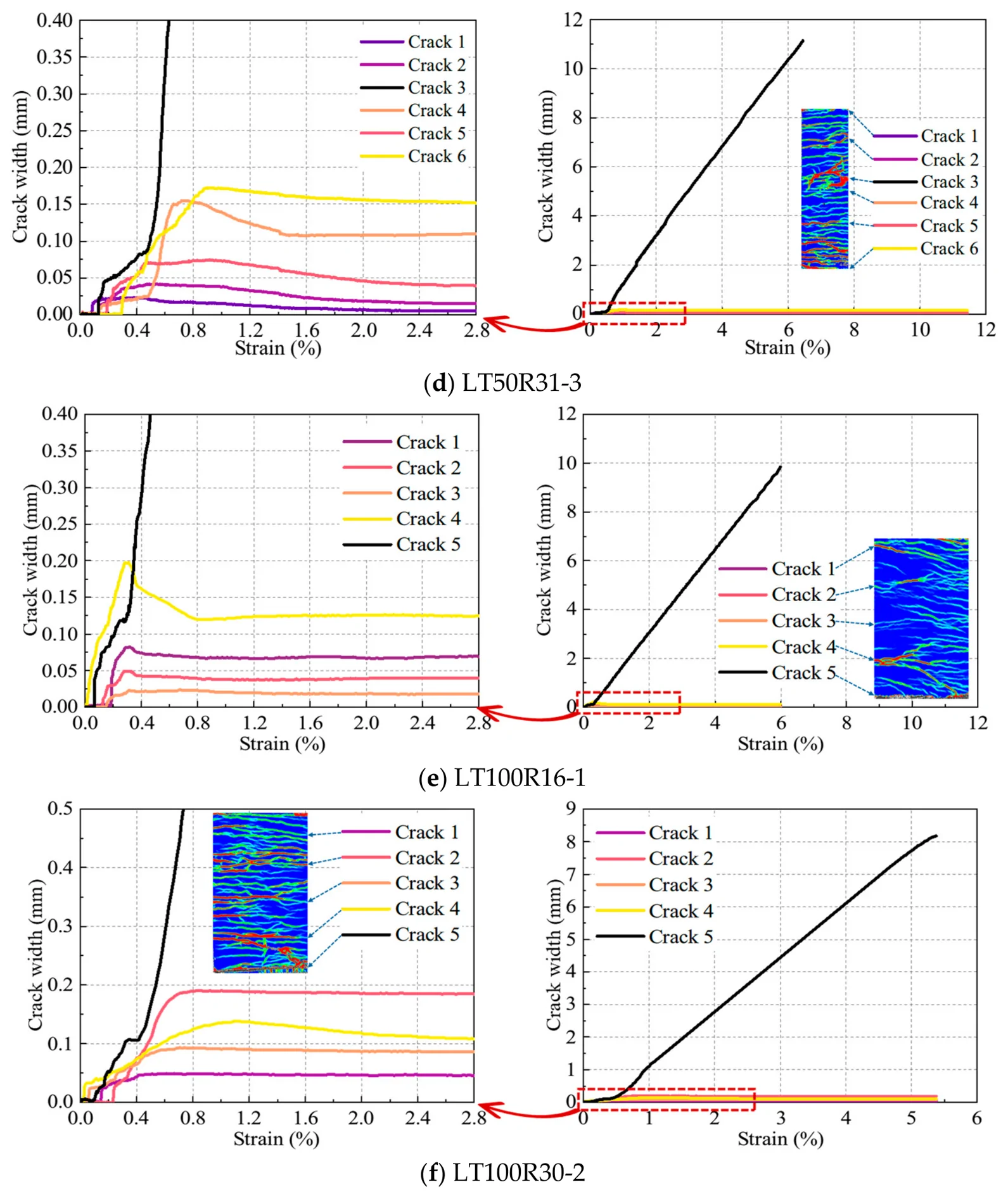
Crack width-strain curves of R-UHPC specimens.

Figure 7 shows the evolution of crack widths in the DIC measurement area for representative R-UHPC tensile specimens with different gauge lengths, thicknesses, reinforcement ratios, and rebar layouts. The strain maps use the same maximum principal strain color scale and numerical range as those in Figure 4, and the black curve in each plot denotes the main crack where final fracture occurred. In specimen ST50R31-3 (Figure 7b), for instance, each crack widened rapidly immediately after initiation. This early rapid opening is attributed to the transfer of tensile stress from the matrix to the bridging fibers, during which the fiber pull-out resistance had not yet fully developed. As the bridging effect of fiber was progressively activated, crack widening was effectively restrained, and all crack openings remained controlled. For strain levels below about 0.4%, the widest crack in the measurement area was not necessarily the one that later developed into the main crack.

Beyond approximately 0.4% strain, strain localization became evident in most specimens: only the width of the main crack increased at an accelerating rate, whereas the widths of secondary cracks gradually decreased and eventually stabilized. The closure of these secondary cracks resulted from the elastic unloading of the UHPC. As steel fibers across the main crack began to pull out extensively, the local bridging capacity degraded, triggering stress redistribution within the specimen. While the steel rebar continued to carry the load, the tensile stress carried by the UHPC decreased, leading to the partial closure of adjacent cracks. This strain localization was eventually observed in all specimens, indicating that the fundamental mechanism of crack-width evolution was not substantially affected by the investigated parameters.

Among these specimens, ST50R11-3 exhibited a more complex crack-width evolution and provides a representative example of how final strain localization can develop through competition among multiple cracked sections. Unlike the typical pattern in which rapid main-crack widening began at approximately 0.4% strain and subsequently developed continuously, ST50R11-3 displayed three distinct accelerations in main-crack widening rate at strains of about 0.4%, 0.8%, and 4.0%, respectively. This behavior implies competitive development among multiple cracked sections.

At approximately 0.4% strain of ST50R11-3, the main crack (Crack 2) suddenly propagated, probably because of the relatively weak post-cracking resistance of its local cracked section. However, its dominance was challenged almost immediately. Cracks 1 and 4 subsequently began to widen rapidly, indicating that the post-cracking resistances of the three cracked sections were sufficiently close for none of them to become immediately dominant. This sequence of rapid widening triggered a competitive deformation process among these three sections. These multiple cracks effectively shared the imposed deformation, contributing to the pronounced strain-hardening characteristics of the specimen. Consequently, the development of secondary cracks restricted the propagation of crack 2, resulting in a plateau in its width evolution between 0.6% and 0.8% strain (Figure 7a), during which the load remained stable without significant softening.

As strain increased to 0.8%, variations in cross-sectional post-cracking strength became decisive. The superior steel fiber bridging effect at the section of Crack 4 led to its stabilization, whereas the bridging effect at Crack 2 peaked and began to decay. This triggered a second rapid increase in the width of Crack 2, accompanied by a noticeable load decline. Subsequently, Crack 2 and Crack 1 expanded synchronously, while the other cracks gradually stabilized or partially closed. The deformation therefore became increasingly concentrated in these two cracked sections, marking the transition from coordinated multiple-crack development to strain localization. Upon reaching a strain of 4.0%, the localization intensified, with deformation concentrating solely at the main crack (Crack 2), while the secondary crack (Crack 1) became dormant.

The evolution of ST50R11-3 therefore illustrates that strain localization in R-UHPC does not necessarily occur immediately after one crack begins to widen rapidly. Instead, several cracked sections can continue to compete for deformation when their post-cracking resistances are comparable, thereby temporarily restraining the localization of any individual crack. Once the bridging resistance of one section begins to degrade relative to the others, this competitive state is disrupted and deformation progressively concentrates at the dominant crack. Thus, although ST50R11-3 followed a more complex crack-width evolution due to the randomness of fiber distribution, its underlying mechanism remained consistent with the general sequence of multiple-crack competition and coordination, main-crack localization, and secondary-crack stabilization observed in the other R-UHPC specimens.

**Figure 8:**
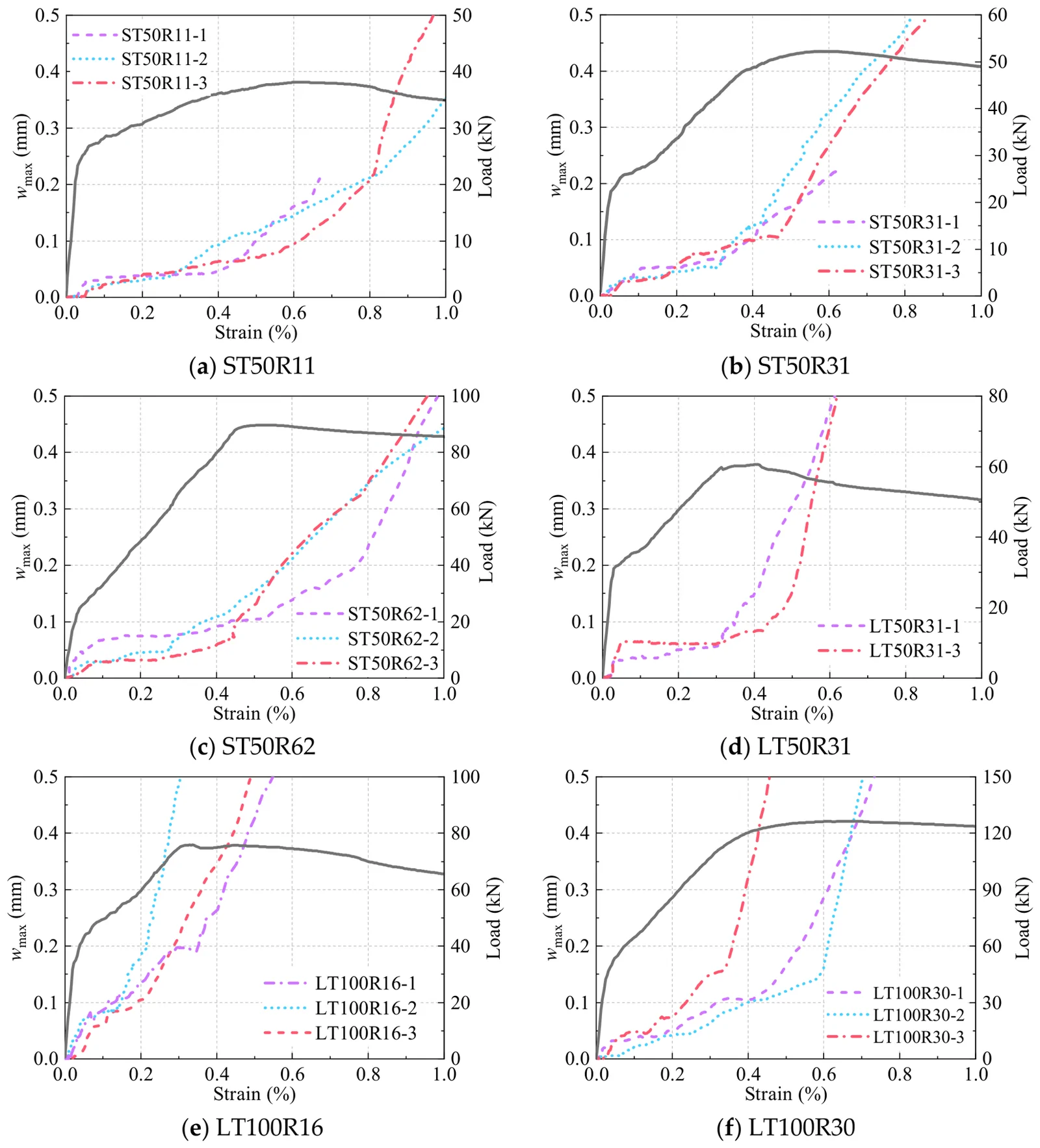
The maximum crack width-strain curves of R-UHPC specimens.

Across all specimens, the development of the maximum crack width (*w*_max_) followed a consistent pattern (Figure 8): rapid opening after initial cracking, slowed growth during the stage of increasing crack count, and a final phase of rapid main-crack widening due to strain localization. The black lines in Figure 8 represent the load–strain curves. LT50R31-2 is not included in Figure 8d because its main crack formed near the edge of the gauge region, where the local speckle quality of this specimen was insufficient for crack-width calculation using DIC. After initial cracking, the maximum crack width increased rapidly until the strain reached approximately 0.05–0.10%, corresponding to the rapid opening of the initial crack (Figure 8a,b). As strain increased from 0.1% to approximately 0.4%, the growth rate slowed noticeably, which coincided with the continuous initiation of secondary cracks. During this phase, the formation of new cracks redistributed tensile stresses, effectively retarding the widening of existing cracks. Consequently, the maximum crack width corresponded to the widest crack among the distributed secondary cracks, rather than to the eventual main crack. Once strain localization developed, the maximum crack width increased almost linearly at a high rate, driven by the rapid opening of the main crack. At this stage, extensive pull-out of steel fibers across the main crack caused the specimen load to reach its peak and subsequently decline.

The effects of gauge length and reinforcement ratio on *w*_max_ were limited within the investigated ranges. A comparison between specimens ST50R31 and LT50R31 with different gauge lengths showed similar maximum crack width–strain relationships before pronounced strain localization, indicating that the maximum crack width was not substantially affected by gauge length. Similarly, the limited effect of reinforcement ratio is evident from a comparison of specimens ST50R11, ST50R31, and ST50R62. Specifically, the average maximum crack widths of these specimens were 0.04 mm, 0.05 mm, and 0.05 mm at 0.2% strain, respectively. These values remained within a narrow range despite the substantial increase in reinforcement ratio from 1.1% to 6.2%.

In contrast, the maximum crack width was strongly affected by specimen thickness when the cross-section was restrained by a single rebar. Compared with specimen LT50R31 with a thickness of 50 mm, specimen LT100R16, which had the same rebar but a thickness of 100 mm, exhibited a much higher growth rate of maximum crack width in the strain range of 0.1–0.4% (Figure 8d,e). At a strain of 0.2%, its maximum crack width reached approximately 0.14 mm, far exceeding the value of 0.05 mm measured for LT50R31 at the same strain. Moreover, LT100R16 also entered the linear rapid-growth stage noticeably earlier. This difference can primarily be attributed to the greater cross-sectional thickness, which places the edge regions of the specimen outside the effective influence zone of the rebar. Consequently, the capacity of the UHPC to distribute cracking is reduced, forcing the total deformation to be accommodated by fewer cracks, thereby leading to a more rapid increase in crack widths.

However, this thickness-related deterioration in crack-width control was substantially mitigated by distributing the reinforcement across the cross-section. A direct comparison between LT100R16 (Figure 8e) and LT100R30 (Figure 8f) shows that the uniformly distributed layout reduced the maximum crack width at 0.2% strain from 0.14 mm to 0.06 mm. The distributed layout extends rebar restraint over a larger portion of the cross-section, thereby reducing the regions outside the effective influence zone and suppressing premature crack localization. This comparison indicates that distributing reinforcement across the cross-section is more effective for crack-width control in thick R-UHPC members than a localized single-rebar layout.

### 3.2. Tensile Response

#### 3.2.1. Load-Strain Curves

**Figure 9:**
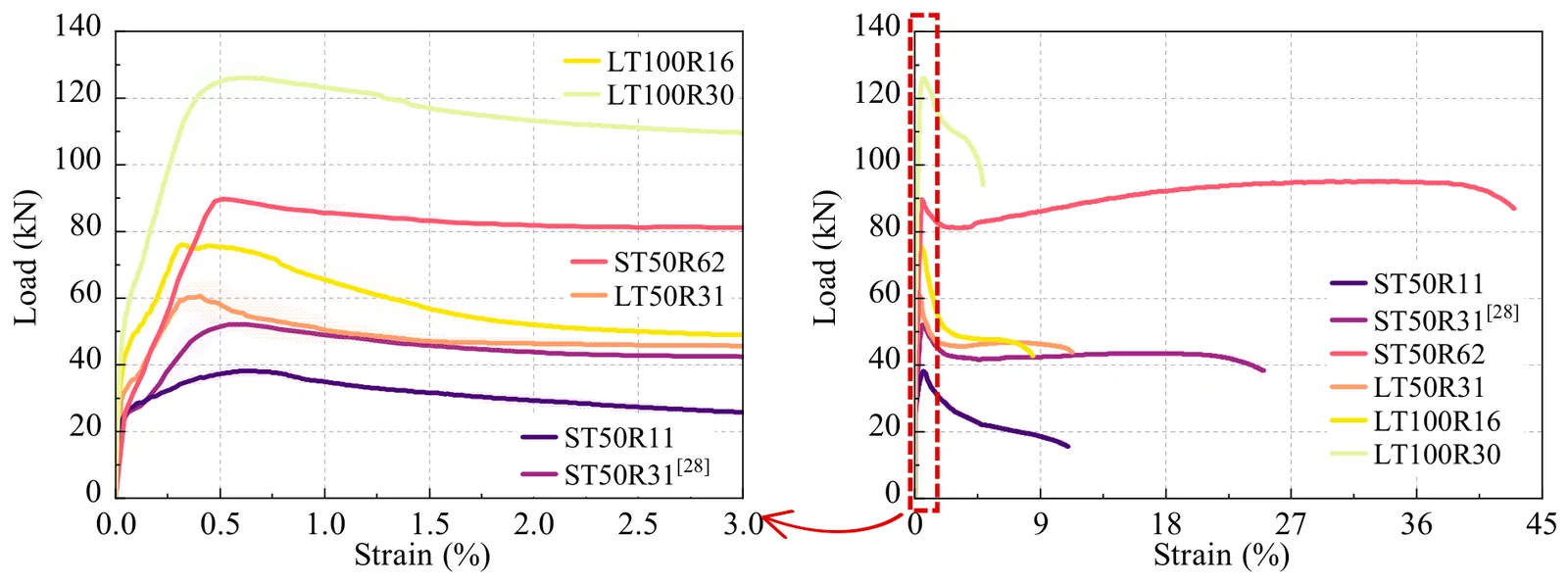
Load-strain response of R-UHPC specimens [28].

Figure 9 presents the average load-strain curves of all 6 R-UHPC specimen groups. Each curve represents the mean result of three specimens with the same parameters, and the shaded bands indicate the corresponding scatter (error bands). Loads and strains at first cracking and the peak load are summarized in Table 4.

Despite the differences in specimen dimensions, reinforcement ratios, and rebar layouts, all specimens exhibited a broadly consistent load-strain evolution up to approximately 3.0% strain. The response was approximately linear elastic until first cracking at about 0.02% strain. Following initial cracking, the stiffness degraded significantly, but the load continued to increase as multiple cracking developed and fiber bridging was progressively mobilized. The first local maximum load, referred to as the peak load herein, generally occurred at approximately 0.3–0.7% strain, after which the load decreased as deformation progressively localized at the main crack and fiber pull-out reduced the tensile contribution of the UHPC. It should be noted that specimen ST50R62-1 exhibited a peak strain of 1.743%. For this specimen, the continued development of two competing cracks before approximately 1.7% strain delayed the formation of the main crack and the subsequent load decrease, resulting in a higher measured peak strain. This evolution is consistent with the crack development described in Section 3.1, indicating that the investigated parameters changed the magnitude and extent of the tensile response without altering the basic transition from multiple cracking to main-crack localization.

Beyond 3.00% strain, the load–displacement curves diverged, allowing the specimens to be classified into two categories. The first category comprises specimens whose curves exhibited a distinct load recovery after the descending branch; for these specimens, the load recovery beyond 3.00% strain was associated with the subsequent tensile resistance provided by the internal rebar, as observed for ST50R31, ST50R62 and LT50R31. For ST50R62, the subsequent second maximum load exceeded the first local maximum load because the strain-hardening of the rebar provided a higher tensile resistance after load recovery. The second category comprises specimens for which the load continued to decrease monotonically until failure without any apparent load recovery in the tensile response, as exemplified by specimen ST50R11, LT100R16 and LT100R30.

A comparison of the curves for ST50R11, ST50R31, and ST50R62 reveals that for specimens featuring a single rebar, increasing the reinforcement ratio from 1.1% to 6.2% leads to a significant enhancement in both the elongation at fracture and load-bearing capacity. However, this monotonic trend was not observed when the rebar layout was simultaneously changed. Specifically, comparing the curves of LT100R16 and LT100R30 shows that although the reinforcement ratio was increased from 1.6% to 3.0% and a distributed rebar layout was adopted, the elongation at fracture declined. These results indicate that the deformation and load-bearing capacities of R-UHPC are jointly affected by reinforcement ratio and rebar layout. Accordingly, the coupled effects of these two parameters should be considered in structural design to provide sufficient deformation capacity and reduce the risk of premature brittle-like failure.

#### 3.2.2. Elongation at Fracture

**Figure 10:**
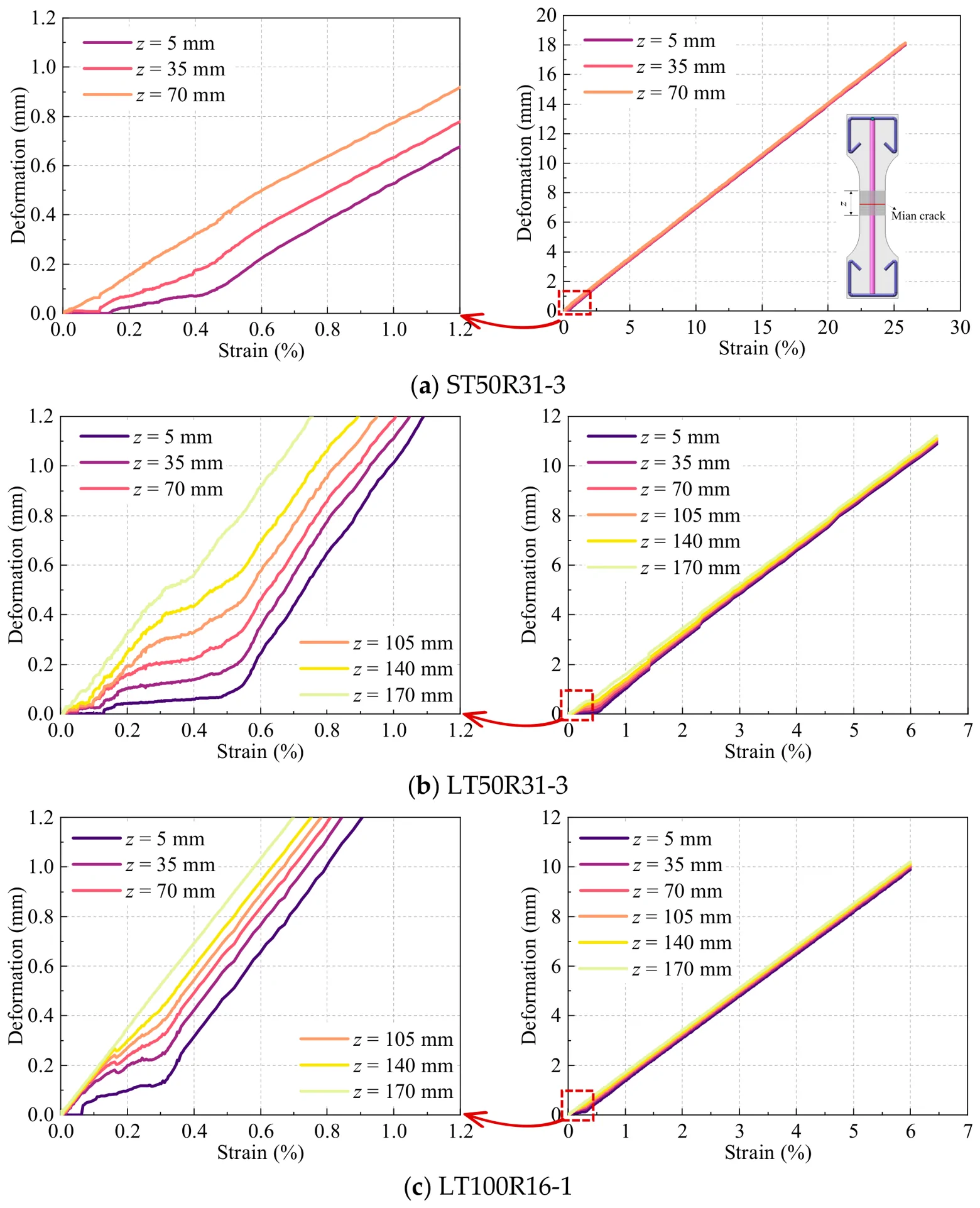
Tensile deformation in the main crack and its vicinity zones (*z*).

Figure 10 illustrates the evolution of tensile deformation in the main crack and its vicinity zones (*z*) with increasing strain for R-UHPC specimens of varying dimensions. Taking specimen ST50R31-3 as an example (Figure 10a), the deformation over the entire gauge length increased linearly with strain from the onset of loading (*z* = 70 mm). In contrast, deformation within the 35 mm vicinity of the main crack showed no significant increase before 0.1% strain, indicating that this region remained uncracked at this stage. Deformation in the 5 mm zone near the main crack began to rise at approximately 0.15% strain, corresponding to the initiation of the main crack, which aligned with the cracking process in Figure 7b.

After reaching about 0.4% strain, the specimen approached its peak load; at this stage, the rate of deformation increases within the 5 mm zone of the main crack accelerated markedly. Subsequently, deformation increments within the 70 mm and 5 mm vicinity of the main crack became almost identical, indicating that overall specimen deformation was fully governed by the widening of the main crack and strain localization became pronounced, with negligible additional deformation occurring elsewhere. At final fracture, accumulated deformation within the 5 mm vicinity of the main crack reached 18.01 mm, accounting for about 99% of the total deformation over the gauge length.

Similar trends were observed in specimens LT50R31 (Figure 10b) and LT100R16 (Figure 10c). However, for specimen LT100R16, the overall deformation became equal to the incremental width of the main crack already at an earlier strain of about 0.3%. This can be attributed to the greater thickness of LT100R16, in which edge regions had already extended beyond the effective influence zone of the rebar. Consequently, crack widening occurred more rapidly, leading to the earlier onset of pronounced strain localization.

Analysis of the deformation evolution within the main crack and its vicinity zones indicates that R-UHPC specimens undergo a stage of distributed tensile deformation before the main crack develops rapidly. In this stage, the rebar and UHPC exhibit coordinated deformation over the tensile region, making the measured specimen strain relatively insensitive to gauge length before the peak load is reached. After rapid opening of the main crack and attainment of the peak load, strain localization becomes pronounced, accompanied by rapid crack widening. The increase in crack width is then almost identical to the increase in overall deformation, confirming that the subsequent tensile deformation is governed by the opening of the main crack. Increasing specimen thickness does not change the basic deformation sequence from distributed tensile deformation to strain localization, but it promotes an earlier onset of strain localization.

The elongations at fracture for specimens ST50R31 and LT50R31 were 27.2% and 11.6%, respectively. However, their absolute tensile deformations at fracture were essentially comparable, measuring 19.04 mm and 19.72 mm. Although the two specimen groups were produced using different UHPC and rebar batches, they exhibited similar crack evolution and nearly identical stabilized crack spacings of 5.98 and 5.99 mm. These observations suggest that gauge length, as a reference measurement length, primarily affects the measured elongation through the averaging effect of localized deformation over different gauge lengths, rather than through changes in the cracking mechanism or strain localization behavior. Consequently, its influence on the strain localization at the main crack is not substantial. Therefore, for R-UHPC specimens with identical cross-sections, the elongation at fracture typically decreases as the gauge length increases.

It should be noted that the elongation at fracture of specimen ST50R31 (27.2%) closely matches the material elongation of the bare rebar (28.1%) when both are measured over a 70 mm gauge length. This correspondence is explained by the mechanism that the post-peak deformation of the R-UHPC specimen primarily stems from the plastic deformation of the rebar within the main crack and its vicinity zone (the strain localization zone), and the total deformation of the rebar in this zone coincides with the fracture deformation of a bare rebar with a 70 mm gauge length. Extending this logic, since the selection of gauge length does not alter the deformation of strain localization zone, the calculated elongation is determined by the geometric relationship between the gauge length and the length of this zone. Specifically, if the gauge length were shorter than the length of the localization zone, the recorded elongation would exceed the material elongation of the bare rebar; conversely, a longer gauge length yields a lower calculated elongation. This variation reflects a geometric scaling effect inherent in the calculation method, not an actual change in the degree of strain localization or the fundamental failure mode.

The section thickness of specimen LT100R16 is twice that of LT50R31, whereas its elongation at fracture is approximately 32.8% lower. This phenomenon is attributed to the fact that increasing the concrete cover thickness or specimen dimensions markedly enhances the lateral confinement effect exerted by the UHPC on the reinforcement [46]. This intensified confinement restricts the expansion of the rebar plastic deformation in the vicinity of the main crack. Consequently, the plastic zone becomes highly localized at the crack section, leading to a lower elongation at fracture despite the use of the same rebar.

#### 3.2.3. Yielded Rebar Length and Deformation Mechanism

**Figure 11:**
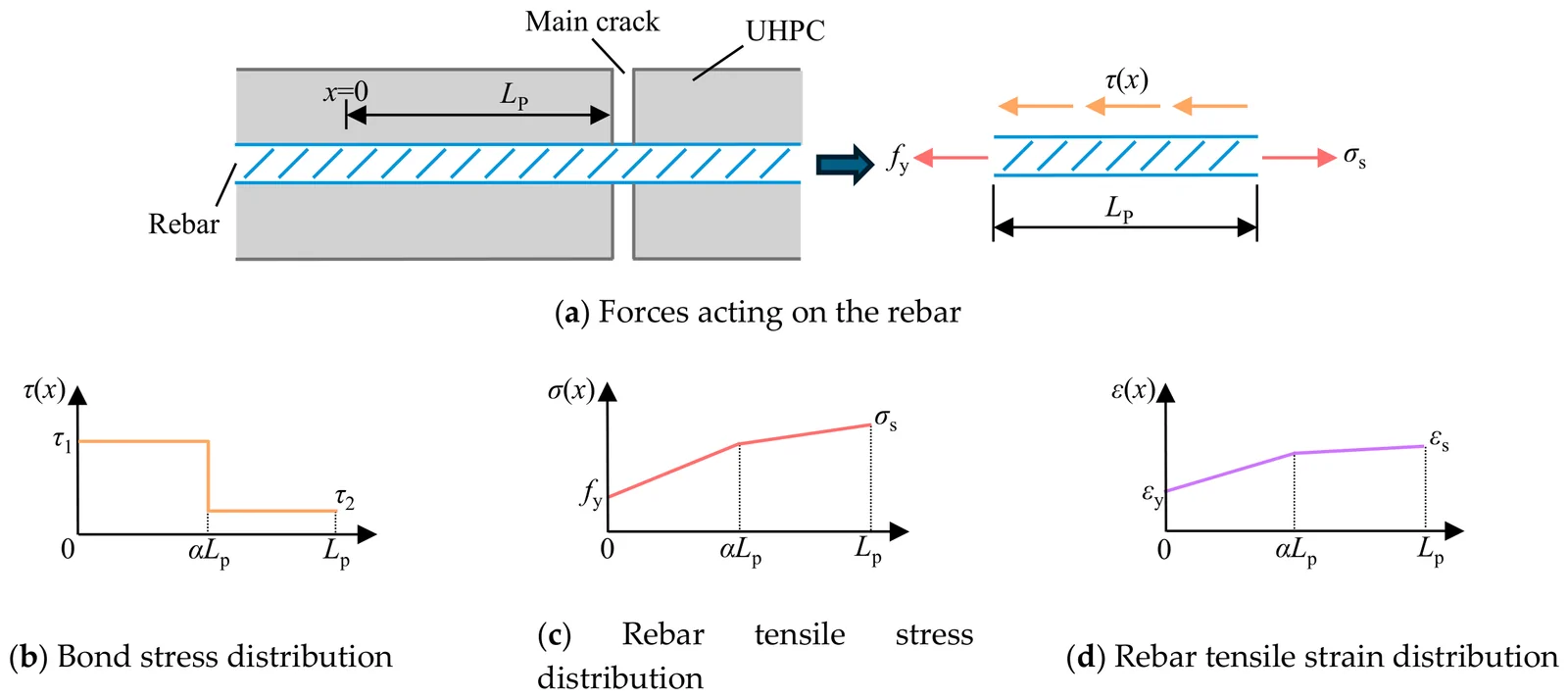
Mechanical analysis of the yielded rebar in the vicinity of the main crack.

Comparing the load-strain responses reveals that rebar configuration governs the elongation at fracture for specimens. To clarify the influence of reinforcement ratio and rebar layout on the elongation at fracture of the specimens and to determine the evolution of elongation at fracture under varying parameters, a mechanical analysis was performed on the rebar in the vicinity of the main crack (Figure 11).

Given that post-peak deformation is predominantly governed by the plastic deformation of the rebar in the vicinity of the main crack, the yielded rebar length *L*_p_ is defined as the distance from the crack face to the rebar stress decreases to its yield strength *f*_y_. Consequently, the rebar within this region resides in the strain-hardening stage (*σ*_s_ > *f*_y_). To analytically determine *L*_p_, the non-uniform interfacial bond stress *τ* distribution is idealized using the simplified model depicted in Figure 11b [47,48].

Based on the force equilibrium of the rebar with a length of *L*_p_ (Figure 11a), the relationship between the rebar tensile stress variation and the interfacial bond stress can be expressed as:

(2) $$\left(\sigma_{s}-f_{y}\right)\cdot A_{s}=\int_{0}^{L_{p}}\tau \left(x\right)\cdot \left(\pi d_{b}\right)\mathrm{dx}$$

where *A*_s_ and *d*_b_ denote the cross-sectional area and diameter of the rebar, respectively. Consequently, *L*_p_ can be derived as:

(3) $$L_{p}=\frac{\left(\sigma_{s}-f_{y}\right)d_{b}}{4\left[\alpha \tau_{1}+\left(1-\alpha \right)\tau_{2}\right]}$$

Upon reaching the peak load, the tensile deformation of the R-UHPC specimen is predominantly attributed to the plastic elongation of the rebar at the main crack and its vicinity. Accordingly, its magnitude is determined by integrating the rebar strain distribution *ε*(*x*) over the yielded rebar length *L*_p_.

(4) $$\begin{array}{l} \frac{\Delta }{2}=\int_{0}^{L_{p}}\varepsilon \left(x\right)\mathrm{dx}=\int_{0}^{L_{p}}\left[\varepsilon_{y}+\frac{\sigma \left(x\right)-f_{y}}{E_{P}}\right]\mathrm{dx}=\varepsilon_{y}L_{p}+\frac{1}{E_{P}}\int_{0}^{L_{p}}\left[\sigma \left(x\right)-f_{y}\right]\mathrm{dx}\\ \;=\left[\varepsilon_{y}+\frac{1-\alpha }{2E_{P}}\left(\sigma_{s}-f_{y}\right)\right]L_{p}+\frac{2\alpha \tau_{1}}{E_{P}d_{b}}{L_{p}}^{2} \end{array}$$

Here, *E*_p_ denotes the plastic modulus, idealized as a constant linear stiffness derived from the measured yield and ultimate points of the rebar. Δ represents the tensile deformation of the R-UHPC specimen at the critical state where the post-peak load-bearing capacity recovers to the ultimate tensile capacity of the internal rebar.

When the rebar stress at the main crack reaches its ultimate strength (*σ*_s_ = *f*_u_), the corresponding yielded rebar length *L*_p_ and the parameter α (Table 5) is determined by solving Equations (3) and (4). At this critical stage, significant rebar slip in the vicinity of the main crack results in the complete loss of chemical adhesion and severe degradation of mechanical interlocking, rendering this region a fully debonded zone (*τ*_2_ ≈ 0 MPa). Conversely, within the interior region where the bond remains relatively intact, the bond stress exhibits a non-uniform distribution that remains largely below the peak strength *τ*_max_. To facilitate analytical derivation, this non-linear bond distribution is idealized as an equivalent uniform bond stress *τ*_1_ [47,48], which is evaluated from the bond–slip responses predicted by the Sturm and Marchand models, respectively:

Sturm model [49]:

(5) $$\tau =\tau_{\max}{\left(\frac{\delta }{\delta_{1}}\right)}^{\beta };\delta <\delta_{1}=\delta_{2}\;\tau_{\max}=\left(0.0018c+0.186\right)f_{c}\;\delta_{1}=\left(-0.0002c+0.001\right)f_{c}+0.0386c+0.0715\;\beta =0.0183d_{b}-0.0055\tau_{\max}+0.304$$

Marchand model [50]:

(6) $$\tau =\tau_{\max}{\left(\frac{\delta }{\delta_{1}}\right)}^{0.4};\delta <\delta_{1}=0.1\mathrm{mm}\;\;\tau =\tau_{\max}=\frac{3.5}{4}\cdot \frac{c}{d_{b}}\sqrt{f_{c}};\delta_{1}\leq \delta <\delta_{2}=0.6\mathrm{mm}$$

where *δ*_1_ and *δ*_2_ denote the slip values, *c* represents the concrete cover thickness. For LT100R16, the minimum cover thickness of 20 mm was adopted in the calculation. and *f*_c_ is the compressive strength of UHPC. Accordingly, the equivalent average bond stress is determined using Equation (7), with the results summarized in Table 5.

(7) $$\tau_{1}=\frac{1}{\delta_{2}}\int_{0}^{\delta_{2}}\tau \left(s\right)\mathrm{ds}$$

Equation (3) explicitly relates the yielded rebar length (*L*_p_) in the vicinity of the main crack and the rebar tensile stress, diameter, and interfacial bond stress. For a given yield strength, as the tensile stress *σ*_s_ approaches the ultimate strength *f*_u_, the progressive expansion of the plastic zone induces a continuous increase in *L*_p_ that reaches its maximum at *σ*_s_ = *f*_u_.

Critically, *L*_p_ exhibits a significant increase with rebar diameter, a phenomenon attributed to the combined effects of force equilibrium requirements and UHPC-rebar interfacial behavior. Fundamentally, based on the axial force analysis (28a), the rebar tensile force scales with the cross-sectional area (∝*d*_b_^2^), whereas the resisting interfacial bond force scales only linearly with the perimeter (∝*d*_b_). Consequently, as *d*_b_ increases, the growth of the resisting bond force lags significantly behind that of the tensile force, necessitating a linear extension of *L*_p_ to maintain force equilibrium. This observation aligns with established findings regarding strain penetration length in plastic hinge regions of reinforced concrete members, reflecting the expansion mechanism of the post-yield plastic zone with increasing diameter [51,52]. Additionally, in thin R-UHPC members, interfacial bond strength typically degrades with increasing diameter [46], further amplifying the demand for *L*_p_ extension.

This mechanism explains the substantial enhancement in elongation observed in 50 mm-thick R-UHPC specimens. As detailed in Table 5, with the reinforcement ratio increased from 1.1% (*d*_b_ = 6 mm) to 6.2% (*d*_b_ = 14 mm), the yielded rebar length (*L*_p_) in the vicinity of the main crack expanded markedly from 27.4 mm to 80.6 mm. This extensive development of rebar plasticity mitigates strain localization, thereby significantly enhancing the elongation at fracture.

For ST50R62, the *L*_p_ calculated using the two bond models were approximately 70.3 and 80.6 mm, which are comparable to or greater than the 70 mm gauge length. This indicates that the plastic deformation zone of the rebar can cover or extend beyond the entire gauge length, resulting in an elongation at fracture of 43.7%, higher than the 31.60% ultimate strain of the bare D14-S rebar measured over the same gauge length. This difference reflects the geometric relationship between the gauge length and the localized plastic deformation region rather than an increase in the intrinsic ductility of the steel rebar.

A further comparison between specimens LT100R16 and LT100R30 reveals that the rebar layout significantly influences the deformation capacity. Despite the latter possessing a reinforcement ratio approximately double that of the former, it exhibited a marked reduction in elongation at fracture. This phenomenon is attributed to the sensitivity of *L*_p_ to the rebar diameter; specifically, the use of smaller diameter rebars in LT100R30 results in a shortening of the yielded rebar length *L*_p_ (Equation (3)), severely constraining the deformation capacity of the specimen. This result reveals that the deformation capacity of R-UHPC members is governed by the rebar configuration (particularly the diameter) rather than the reinforcement ratio magnitude alone. Consequently, achieving enhanced ductility in R-UHPC structural design requires optimizing the rebar diameter, extending beyond a simple increase in the reinforcement ratio.

**Figure 12:**
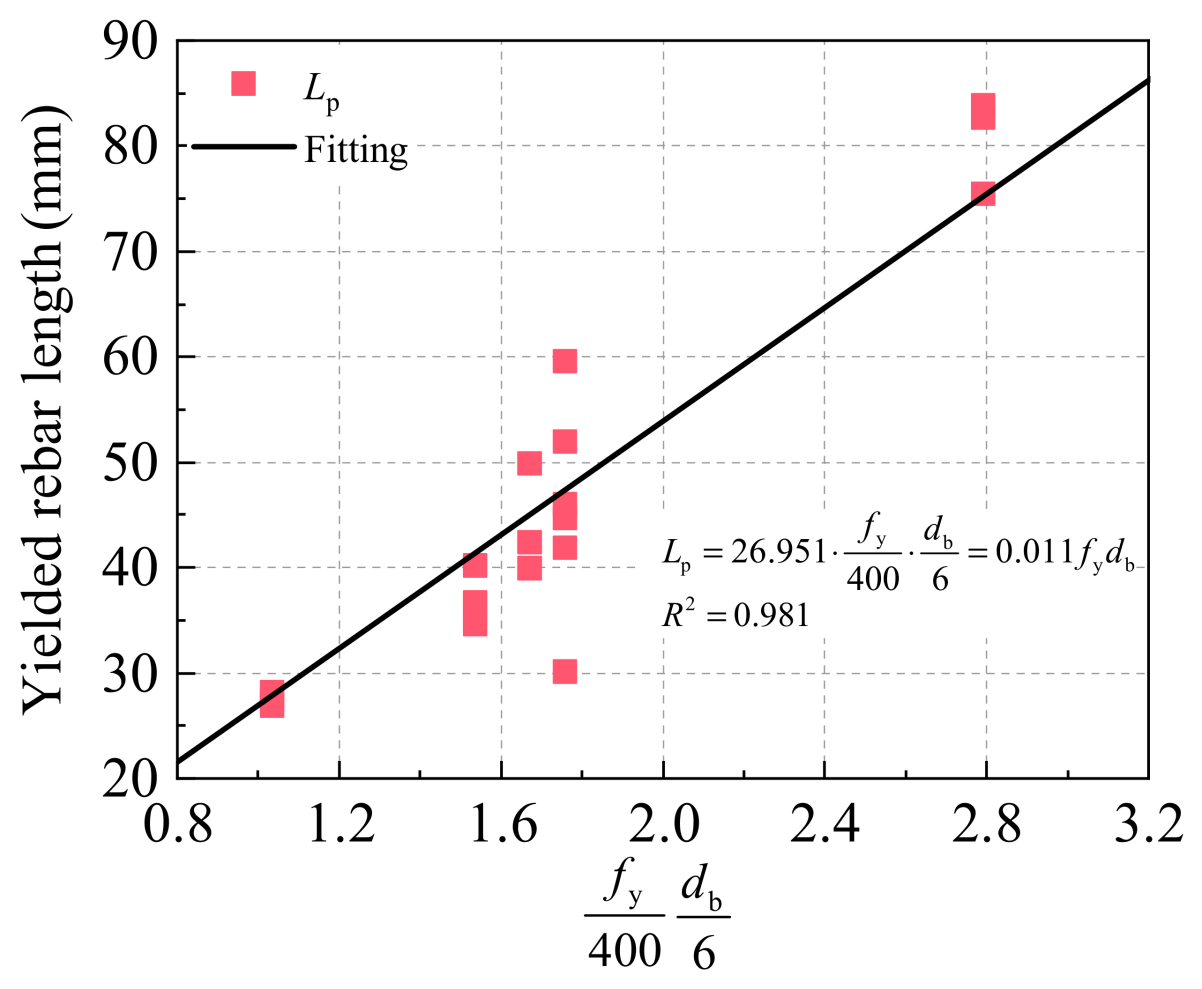
Yielded rebar length *L*_p._

As illustrated in Figure 12, normalized parameters $\frac{f_{y}}{400}$ and $\frac{d_{b}}{6}$ were employed to analyze the calculated *L*_p_ data. and the obtained *R*^2^ reflects the fitting quality of the regression relationship rather than experimental validation The regression coefficients obtained from the two bond models were 0.010 and 0.011 for the Sturm and Marchand models, respectively. Considering that the Marchand model predicts a slightly longer yielded rebar length *L*_p_, which corresponds to a relatively larger plastic deformation region, the following regression relationship based on the Marchand model was adopted:

(8) $$L_{p}=26.951\cdot \frac{f_{y}}{400}\cdot \frac{d_{b}}{6}=0.011f_{y}d_{b}$$

Notably, the derived coefficient of 0.011 is approximately half the empirical value of 0.022 widely established for strain penetration in the plastic hinge regions of conventional reinforced concrete members [52]. This significant reduction underscores the superior interfacial bond performance of UHPC, which effectively limits the extension of the yielded rebar length. Consequently, the proposed model not only quantifies *L*_p_ and characterizes the extent of strain localization in R-UHPC, but also provides a fundamental basis for understanding the mechanism of deformation capacity in R-UHPC members.

### 3.3. Tensile Strength Reduction of the UHPC Component in R-UHPC

For tensioned R-UHPC specimens under tension, the peak load can be decomposed into the contribution of the rebar and the UHPC component. Based on existing studies on the tensile capacity of R-UHPC [29], the peak load is expressed as:

*N*_T_ = *μf*_c,t_*A*_u_ + *f*_s,y_*A*_s_ (9)

where *N*_T_ is the peak load of the R-UHPC specimen; *A*_u_ and *A*_s_ are the cross-sectional areas of the UHPC component and steel reinforcement, respectively; *f*_c,t_ is the tensile strength of plain UHPC; *f*_s,y_ is the yield strength of the steel rebar; and *μ* is the strength reduction factor of the UHPC component.

To quantify the tensile contribution of the UHPC component at peak load, the effective tensile strength of the UHPC component ($f_{c,t}^{e\mathrm{ff}}$) is defined as the average tensile stress carried by UHPC after subtracting the reinforcement yielding contribution from the total peak load:

(10) $$f_{c,t}^{e\mathrm{ff}}=(N_{T}-f_{s,y}A_{s})/A_{u}$$

(11) $$\mu =f_{c,t}^{e\mathrm{ff}}/f_{c,t}$$

*μ* characterizes the effective tensile contribution of the UHPC component in R-UHPC, calculated by the peak load listed in Table 4. For example, for LT100R16, *N*_T_ = 79.7 kN, *f*_s,y_ = 422 MPa, *A*_s_ 78.54 mm^2^, *A*_u_ = 4921.46 mm^2^, and *f*_c,t_ = 13.7 MPa. According to Equation (10), the effective tensile strength of the UHPC component is *f*_c,t_^eff^ = (79.7 × 10^3^ − 422 × 78.54)/4921.46 = 9.46 MPa. Thus, Equation (11) gives *μ* = 9.46/13.7 = 0.69. Based on this procedure, the mean strength reduction factors of the UHPC component were calculated as 0.99, 0.82, 0.53, 0.84, 0.69, and 0.86 for specimens ST50R11, ST50R31, ST50R62, LT50R31, LT100R16, and LT100R30, respectively.

Comparison of the strength reduction factors of the UHPC component in specimens ST50R31, LT50R31, and LT100R30 shows that these specimens exhibited nearly identical reduction factors, with values of 0.82, 0.84, and 0.86, respectively. This strength reduction can be primarily attributed to the presence of rebar, which disturbs the rheological behavior and spatial distribution of steel fibers. This disruption impairs both the uniformity of fiber distribution and the fiber orientation. Furthermore, during tension, the relative slip between the steel rebars and the UHPC induces stress concentrations near the transverse ribs of the rebars. This leads to the initiation of local microcracks and exacerbates internal damage, thereby limiting the effective tensile contribution of the UHPC component. This consistency suggests that for a reinforcement ratio of approximately 3.1%, the effective tensile strength of the UHPC component can be conservatively taken as 0.82 times its material tensile strength. This value is comparable to the findings from existing studies with similar reinforcement ratios [53] and provides an important reference for tensile performance analysis and load-bearing capacity assessment of R-UHPC specimens.

**Figure 13:**
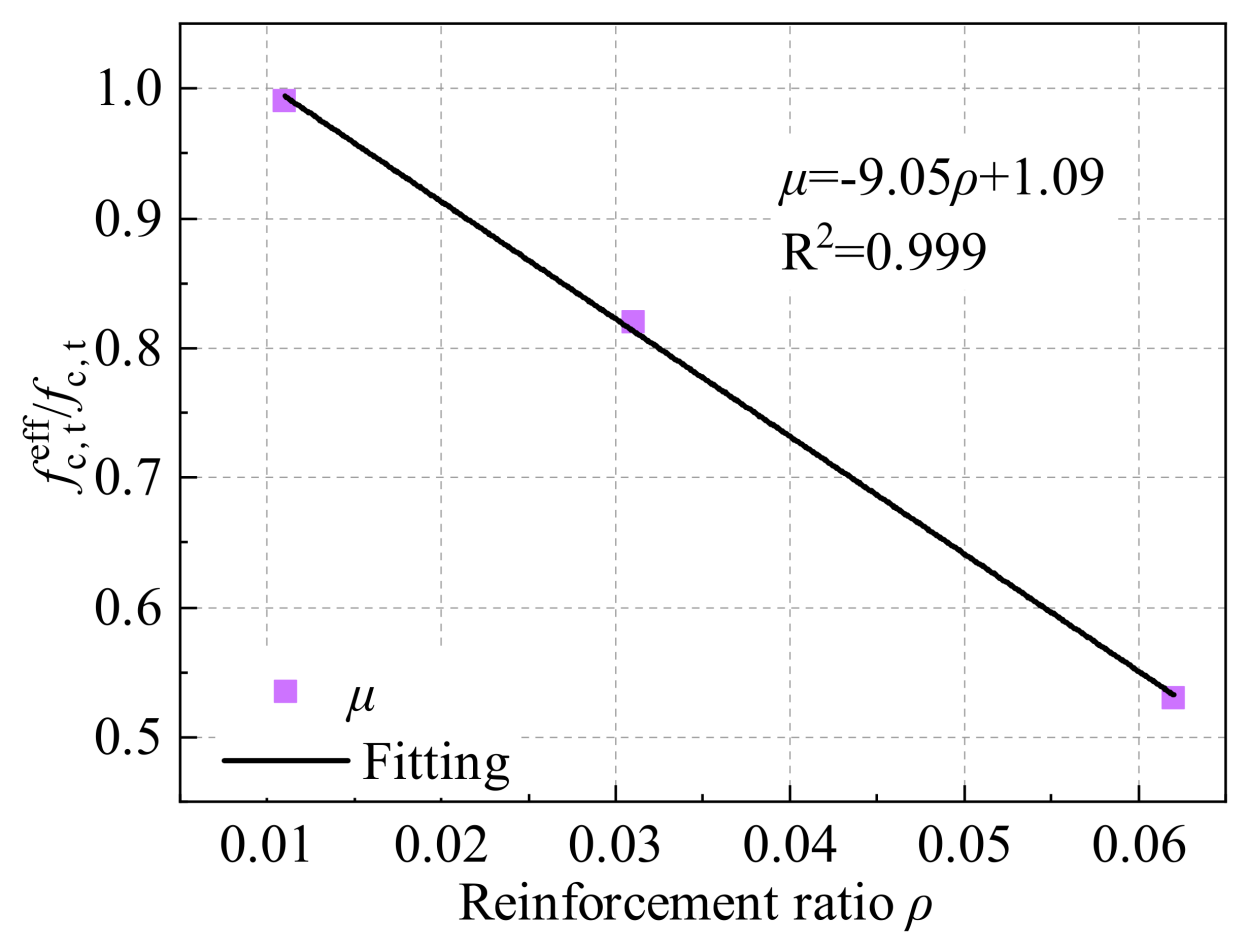
Strength reduction factor (*μ*) of the UHPC component.

For R-UHPC specimens with an identical thickness of 50 mm, the strength reduction factor of UHPC component showed a clear dependence on reinforcement ratio (Figure 13). At a reinforcement ratio of 1.1%, ST50R11 exhibited a strength reduction factor of 0.99, indicating that the intrinsic tensile strength of UHPC was almost fully mobilized. This factor decreased to 0.82 and 0.53 for ST50R31 and ST50R62, respectively, as the reinforcement ratio increased to 3.1% and 6.2%. Such a decrease is mainly associated with the greater disturbance imposed by rebars on steel-fiber dispersion and orientation within the UHPC matrix at higher reinforcement ratios. A higher reinforcement ratio may promote local regions with reduced fiber content or disordered fiber alignment in the vicinity of rebars, thereby reducing the crack-bridging efficiency of the fibers. Conversely, a lower reinforcement ratio mitigates these adverse effects and, together with the increased relative cover thickness, can partially suppress localized damage around the transverse ribs during interfacial slip and reduce the associated stress concentration. Consequently, reducing the reinforcement ratio enables a larger fraction of the intrinsic tensile capacity of UHPC to be mobilized in R-UHPC specimens.

When the specimen thickness increased to 100 mm, the apparent strength contribution of the UHPC component decreased further. For specimen LT100R16, the effective tensile strength of the UHPC component was only 0.69 times that of plain UHPC, showing a larger reduction than that observed in the 50 mm-thick specimens. This reduction may be related to the limited restraint provided by the rebar in the thicker cross-section. Due to the increased distance between the rebar and specimen edges, the restraint effect of the rebar may become insufficient in edge regions, which may contribute to non-uniform cracking and strain localization.

**Figure 14:**
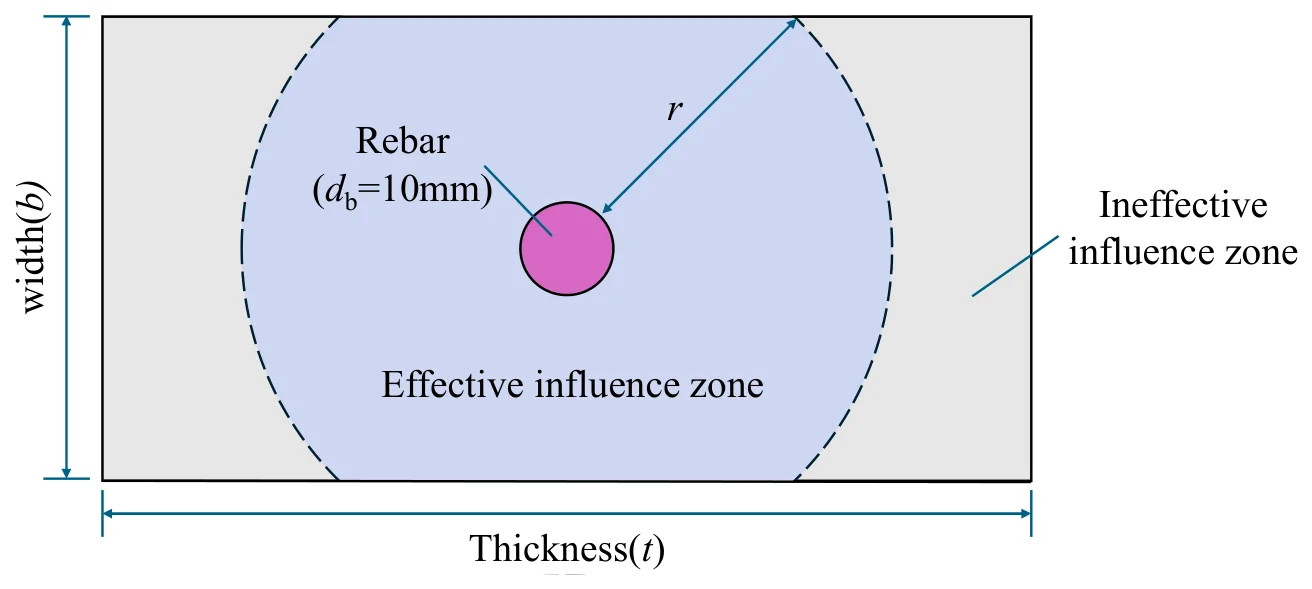
The effective influence zone of rebar.

To quantify this spatially limited restraint, an effective influence zone around the rebar was introduced, as illustrated in Figure 14. The UHPC within this zone was estimated with reference to the range of μ obtained from the 50 mm-thick specimens with similar reinforcement ratio, whereas the tensile contribution of UHPC outside this zone was neglected at the peak load. The effective influence zone was represented geometrically by the circular region shown in Figure 14. When this circular region intersects the two lateral boundaries of a rectangular section but does not reach the other two boundaries, the effective UHPC area can be expressed as:

*F*_UHPC_ = *f*_c,t_ × *μ* × *A*_eff_ (12)

(13) $$A_{\mathrm{eff}}=2{\left(r+\frac{d_{b}}{2}\right)}^{2}\arcsin\left(\frac{b}{2r+d_{b}}\right)+b\sqrt{{\left(r+\frac{d_{b}}{2}\right)}^{2}-\frac{b^{2}}{4}}-\frac{\pi d_{b}^{2}}{4}$$

where *A*_eff_ represents the cross-sectional area of the UHPC within the effective influence zone of the rebar; *F*_UHPC_ denotes the tensile load resisted by the UHPC component within the effective influence zone; and *r* refers to the effective influence radius measured from the rebar surface; *d*_b_ is the rebar diameter, and *b* is the section width.

A range of μ = 0.85–1.00, corresponding to the values observed in the 50 mm-thick specimens (Figure 13), was considered to estimate the possible range of the effective influence radius. The resulting effective influence radius ranged from 31.4 to 41.6 mm, corresponding to approximately 3.1–4.2*d*_b_. Previous studies on UHPC–rebar bond behavior have reported that the influence of cover thickness on bond strength tends to stabilize at approximately 3–4 *d*_b_ [50,54]. The effective radius estimated under the adopted assumption (3.1–4.2 *d*_b_) is comparable to this reported range. Combined with the pronounced strain localization observed by DIC in the edge regions of LT100R16, these results suggest that the limited rebar influence range should be considered when evaluating the tensile contribution of the UHPC component in thick R-UHPC sections.

Further comparison between specimens LT100R30 and LT100R16, which feature distinct rebar layout, reveals that a rational rebar layout is vital for enhancing the efficiency of the UHPC component contribution. Experimental results demonstrate that the effective tensile strength of the UHPC component in specimen LT100R30 (with three distributed rebars) significantly higher than specimen LT100R16 (with a single central bar). This disparity arises because the distributed layout ensures that the effective influence zones cover the cross-section comprehensively, thereby maximizing the load-bearing potential of the UHPC component.

## 4. Conclusions

This paper investigates the measured tensile response and cracking behavior of R-UHPC dog-bone specimens under quasi-static direct tension, focusing on the effects of thickness (50 and 100 mm), gauge length (70 and 170 mm), reinforcement ratio (1.1–6.2%), and rebar layout (a single rebar and distributed rebars). The investigated specimens employed a commercial UHPC with a steel fiber volume fraction of 2.0% and HRB400 steel rebar. Within these experimental conditions, the main findings are summarized as follows:

1. Before rapid main-crack propagation, which approximately coincides with the peak load, tensile deformation in the R-UHPC specimens remains distributed over the full gauge length, with multiple cracks opening progressively. The post-peak behavior is dominated by strain localization, with deformation concentrating primarily at the main crack. When deformation becomes localized, increasing the gauge length is associated with a lower measured elongation at fracture, as the localized deformation is averaged over a longer measurement base.
2. For R-UHPC specimens with the same dimension, the length of the yielded rebar region (*L*_p_) in the vicinity of the main crack is predominantly governed by the rebar diameter. As the diameter increases, the increase in bond force lags behind that of the tensile force, necessitating an extended *L*_p_ to maintain force equilibrium, effectively mitigating strain localization and enhancing the overall elongation at fracture.
3. For the 100 mm-thick specimens with single rebar layout in this study, the edge regions extended beyond the effective influence zone of the rebar. These edge regions exhibited intensified strain localization and cross-sectional strain non-uniformity, contributing to a reduction in the effective tensile strength of the UHPC component to approximately 0.69 times that of plain UHPC.
4. If the UHPC component of specimens is entirely within the effective influence zone of rebar, the reinforcement ratio dictates the magnitude of effective tensile strength reduction in the UHPC component. A reinforcement ratio of 1.1% retains this strength at 0.99 times that of plain UHPC; increasing the reinforcement ratio to 3.1% and 6.2% reduces this value to 0.82 and 0.53, respectively. The assessment of R-UHPC load-bearing capacity is suggested to take into account the UHPC strength detection based on the reinforcement ratio.
5. Compared with a concentrated single rebar configuration, the distributed rebar configuration for thick specimens investigated in this study exhibited an enlarged effective influence zone, improved crack control and enhanced UHPC component strength. At the same time, the associated reduction in rebar diameter in distributed rebar layout contributed to intensified strain localization and decreased elongation at fracture.

Future study is suggested to include parameters, such as UHPC mixtures, steel fiber contents, rebar grades and cross-sectional scales, and also enlarged scale.

## Figures and Tables

**Table 1:** Specimen details.

Group	Thickness *t* (mm)	Specimen Length *L* (mm)	Gauge Length *l* (mm)	Rebar Diameter *d*_b_ (mm)	Number of Rebars *n*	Reinforcement Ratio *ρ* (%)
ST50R11	50	360	70	6	1	1.1
ST50R31 [28]	50	360	70	10	1	3.1
ST50R62	50	360	70	14	1	6.2
LT50R31	50	500	170	10	1	3.1
LT100R16	100	500	170	10	1	1.6
LT100R30	100	500	170	8	3	3.0

**Table 2:** Mechanical properties of UHPC samples.

UHPC Group	Number of Specimens	Fiber Content (vol%)	Tensile Strength *f*_t_ (MPa)	Compressive Strength *f*_c_ (MPa)	Elastic Modulus *E* (GPa)
UHPC-S	3	2.0	10.9 ± 0.2	156.1 ± 3.5	46.1 ± 2.4
UHPC-L	3	2.0	13.7 ± 0.1	167.3 ± 3.3	46.4 ± 1.0

Notes: UHPC-S and UHPC-L denote the specific casting batches used for the 360 mm and 500 mm length specimens, respectively. The values are presented as mean ± standard deviation based on 3 specimens.

**Table 3:** Mechanical properties of steel rebar.

Rebar Group	Diameter *D* (mm)	Elastic Modulus *E* (GPa)	Yield Strength *f*_y_ (MPa)	Ultimate Strength *f*_u_ (MPa)	Ultimate Strain at 70 mm Gauge Length (%)	Ultimate Strain at 170 mm Gauge Length (%)
D6-S	6	219	414	650	20.07	14.07
D10-S	10	220	401	549	28.11	19.71
D14-S	14	202	479	653	31.60	22.16
D8-L	8	204	461	667	21.76	15.26
D10-L	10	202	422	601	27.62	19.37

Notes: The suffixes ‘-S’ and ‘-L’ denote the specific rebar batches used for the 360 mm and 500 mm length specimens, respectively.

**Table 4:** Tensile Results of R-UHPC.

Specimens	Cracking Load (kN)	Cracking Strain (%)	Peak Load (kN)	Peak Strain (%)	Elongation at Fracture (%)
ST50R11-1	23.9	0.029	38.8	0.609	11.1
ST50R11-2	21.8	0.024	36.3	0.619	11.6
ST50R11-3	23.6	0.029	39.6	0.670	11.0
Avg. ± S.D.	23.1 ± 0.9	0.027 ± 0.002	38.3 ± 1.4	0.633 ± 0.027	11.2 ± 0.3
ST50R31-1	16.5	0.024	51.5	0.621	26.3
ST50R31-2	20.3	0.026	55.7	0.398	29.4
ST50R31-3	30.3	0.029	52.4	0.517	25.8
Avg. ± S.D.	22.37 ± 5.8	0.026± 0.002	53.2 ± 1.8	0.512 ± 0.091	27.2 ± 1.6
ST50R62-1	15.9	0.034	82.5	1.743	43.0
ST50R62-2	20.0	0.021	91.8	0.514	44.9
ST50R62-3	19.4	0.031	87.7	0.505	43.1
Avg. ± S.D.	18.4 ±1.8	0.029 ± 0.006	87.3 ± 3.8	0.921 ± 0.581	43.7 ± 0.9
LT50R31-1	29.9	0.024	62.2	0.314	13.5
LT50R31-2	30.9	0.030	55.9	0.342	9.8
LT50R31-3	27.7	0.023	64.9	0.417	11.4
Avg. ± S.D.	29.5 ± 1.3	0.026 ± 0.003	61.0 ± 3.8	0.358 ± 0.043	11.6 ± 1.5
LT100R16-1	32.8	0.017	77.7	0.494	8.6
LT100R16-2	23.8	0.018	77.6	0.450	8.7
LT100R16-3	33.2	0.015	83.7	0.307	6.0
Avg. ± S.D.	30.0 ± 4.4	0.016 ± 0.001	79.7 ± 2.9	0.417 ± 0.080	7.8 ± 1.3
LT100R30-1	41.9	0.014	132.6	0.595	6.4
LT100R30-2	17.7	0.020	123.5	0.657	5.1
LT100R30-3	40.1	0.014	123.5	0.701	5.0
Avg. ± S.D.	33.2 ± 11.0	0.016 ± 0.003	126.5 ± 4.3	0.651 ± 0.043	5.5 ± 0.6

Note: Avg. and S.D. are abbreviations for average and standard deviation, respectively. Peak load represents the first local maximum load before the subsequent load recovery.

**Table 5:** Yielded rebar length *L*_p._

Specimens	Δ (mm)	Sturm Model			Marchand Model		
		*τ*_1_ (MPa)	*α* (%)	*L*_p_ (mm)	*τ*_1_ (MPa)	*α* (%)	*L*_p_ (mm)
ST50R11-1	5.8	28.9	42.6	28.8	38.2	33.9	27.3
ST50R11-2	5.7		43.2	28.4		34.4	26.9
ST50R11-3	6.0		41.5	29.6		33.0	28.1
Avg. ± S.D.	5.8 ± 0.1		42.4 ± 0.7	28.9 ± 0.5		33.8 ± 0.6	27.4 ± 0.5
ST50R31-1	11.2	26.7	34.3	40.4	20.8	42.0	42.3
ST50R31-2	13.7		28.9	47.9		35.7	49.8
ST50R31-3	10.4		36.4	38.0		44.5	39.9
Avg. ± S.D.	11.8 ± 1.4		33.2 ± 3.2	42.1 ± 3.2		40.7 ± 3.7	44.0 ± 4.2
ST50R62-1	19.7	24.8	37.7	65.1	13.4	60.3	75.4
ST50R62-2	22.8		33.4	73.4		54.3	83.8
ST50R62-3	22.4		33.9	72.4		55.0	82.7
Avg. ± S.D.	21.6 ± 1.4		35.0 ± 1.9	70.3 ± 3.7		56.5 ± 2.7	80.6 ± 3.7
LT50R31-1	15.6	29.0	27.2	56.9	21.6	34.8	59.5
LT50R31-2	10.0		39.4	39.2		49.6	41.8
LT50R31-3	13.2		31.3	49.3		39.9	51.9
Avg. ± S.D.	13.4 ± 2.9		32.6 ± 5.1	48.5 ± 7.2		41.4 ± 6.1	51.1 ± 7.2
LT100R16-1	11.3	29.0	35.7	43.3	21.6	45.1	45.9
LT100R16-2	10.9		36.8	42.1		46.4	44.7
LT100R16-3	6.3		56.2	27.5		68.8	30.1
Avg. ± S.D.	9.5 ± 2.3		42.9 ± 9.4	37.6 ± 7.2		53.4 ± 10.9	40.2 ± 7.2
LT100R30-1	7.7	28.7	37.9	37.9	21.6	47.4	40.2
LT100R30-2	6.8		41.9	34.3		52.1	36.6
LT100R30-3	6.3		44.5	32.3		55.1	34.6
Avg. ± S.D.	6.9 ± 0.6		41.4 ± 2.7	34.8 ± 2.3		51.5 ± 3.2	37.1 ± 2.3

Note: Avg. and S.D. are abbreviations for average and standard deviation, respectively.

## Data Availability

The original contributions presented in this study are included in the article. Further inquiries can be directed to the corresponding author.
